# Mean arterial pressure to norepinephrine equivalent dose ratio for predicting renal replacement therapy requirement: a retrospective analysis from the MIMIC-IV

**DOI:** 10.1007/s11255-023-03908-3

**Published:** 2024-01-18

**Authors:** Qiang Liu, Yawen Fu, Zhuo Zhang, Ping Li, Hu Nie

**Affiliations:** 1 Department of Emergency, West China Hospital of Sichuan University, Chengdu, Sichuan China; 2 West China Xiamen Hospital of Sichuan University, Xiamen, Fujian China

**Keywords:** Mean arterial pressure, Norepinephrine equivalent dose, Acute kidney injury, Renal replacement therapy, MIMIC-IV

## Abstract

**Background:**

This study aimed to assess the predictive value of the ratio of mean arterial pressure (MAP) to the corresponding peak rate of norepinephrine equivalent dose (NEQ) within the first day in patients with shock for the subsequent renal replacement therapy (RRT) requirement.

**Methods:**

Patients were identified using the Medical Information Mart for Intensive Care IV (MIMIC-IV) database. The relationship was investigated using a restricted cubic spline curve, and propensity score matching(PSM) was used to eliminate differences between groups. Odds ratios (OR) with 95% confidence intervals (CI) were calculated using logistic regression. Variable significance was assessed using extreme gradient boosting (XGBoost), and receiver operating characteristic (ROC) curves were generated.

**Results:**

Of the 5775 patients, 301 (5.2%) received RRT. The MAP/NEQ index showed a declining L-shaped relationship for RRT. After PSM, the adjusted OR per 100 mmHg/mcg/kg/min for RRT was 0.93(95% CI 0.88–0.98). The most influential factors for RRT were fluid balance, baseline creatinine, and the MAP/NEQ index. The threshold for the MAP/NEQ index predicting RRT was 161.7 mmHg/mcg/kg/min (specificity: 65.8%, sensitivity: 74.8%) with an area under the ROC curve of 75.9% (95% CI 73.1–78.8).

**Conclusions:**

The MAP/NEQ index served as an alternative predictor of RRT necessity based on the NEQ for adult patients who received at least one vasopressor over 6 h within the first 24 h of intensive care unit(ICU) admission. Dynamic modulation of the MAP/NEQ index by the synergistic use of various low-dose vasopressors targeting urine output may be beneficial for exploring individualized optimization of MAP.

**Supplementary Information:**

The online version contains supplementary material available at 10.1007/s11255-023-03908-3.

## Introduction

Vasopressors are commonly used to maintain mean arterial pressure(MAP) in the intensive care unit (ICU). This practice is strongly correlated with acute kidney injury (AKI), which is estimated to occur in 30–60% of adult patients. AKI is deemed a heterogeneous syndrome that significantly increases the risk of adverse kidney events such as the need for renal replacement therapy (RRT), development of chronic kidney disease (CKD), and mortality [1].

Previous studies have suggested that the mean perfusion pressure (MPP), calculated as the difference between the MAP and central venous pressure (CVP), ≤ 60 mmHg is linked to the onset and progression of AKI [2–4]. Nonetheless, other studies have indicated a significant relationship between decreased MAP and renal injury in the ICU, suggesting the need to avoid severe hypotension [5, 6]. Determining the optimal MAP to reduce the risk of AKI requires individualized adjustments based on each patient's unique circumstances [7, 8].

Hemodynamic instability often causes renal dysfunction due to insufficient perfusion and impaired kidney microcirculation [9]. Different vasopressors exert their effects by selectively binding to their corresponding receptors, modulating the renal perfusion pressure and microcirculation. Thus, quantification of vasopressor exposure is essential for comparative analyses in clinical trials and scholarly discourse. Several conversion formulas have been proposed to address the issue of norepinephrine equivalent dose (NEQ) [10–12].

Bosch et al. proposed the MAP/NEQ index as a metric for assessing the severity of septic shock, taking inspiration from the oxygenation index used to evaluate respiratory distress. This novel approach enhanced prognostic efficacy compared to the conventional sepsis severity score [13]. Furthermore, Wang et al. demonstrated that the MAP/NEQ index could serve as a valuable indicator for determining the optimal timing to initiate enteral nutrition in shock patients [14].

The criteria for initiating RRT remain debatable. Early identification and intervention are crucial before these conditions escalate to the critically severe stages. Therefore, we conducted a retrospective study to investigate whether the MAP/NEQ index can be an assessment tool for evaluating RRT requirement and sustained renal dysfunction.

## Materials and methods

### Study design and participants

**Fig. 1:**
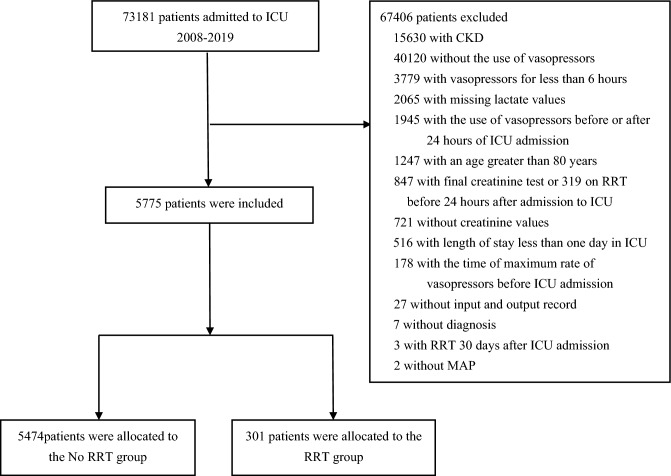
Flowchart of patient selection *CKD* chronic kidney disease, *ICU* intensive care unit, *RRT* renal replacement therapy, *MAP* mean arterial pressure

This retrospective study used a publicly accessible critical care database, the Medical Information Mart for Intensive Care IV (MIMIC-IV) [15, 16]. Published by the Computational Physiology Laboratory at the Massachusetts Institute of Technology, the MIMIC-IV (v2.2) encompassed comprehensive data on patients admitted to the ICU at the Beth Israel Deaconess Medical Center between 2008 and 2019. Our research focused on adult patients who received at least one vasopressor over 6 h within the first 24 h of ICU admission, as shown in Fig. 1 [17]*.* These exclusions were: patients with pre-existing CKD, patients with lack of administration of vasopressors or administration of vasopressors without 24 hours or duration of vasopressors less than 6 h, patients with the time of maximum rate of vasopressors before ICU admission, patients with RRT initiation or last creatinine test within 24 h or beyond 30 days, patients with an ICU stay that were less than one day or with age over 80 years, patients who lacked crucial information such as diagnosis, MAP, input, output, creatinine, and lactate. However, patients who received RRT and whose last plasma creatinine level within 30 days did not exceed two times the baseline were excluded for sustained renal dysfunction. Our data collection included demographics, MAP, baseline creatinine, and comorbidities. Throughout the course of treatment, we collected the following variables: indicators of disease severity (duration of MAP < 65 mmHg, utilization of invasive mechanical ventilation, platelet, lactate, and maximum chloride), administration of vasopressors (norepinephrine, epinephrine, vasopressin, phenylephrine, and dopamine), use of inotropic support(dobutamine and milrinone), administration of potentially nephrotoxic drugs (vancomycin), RRT and variables that may be pertinent to outcomes (liquid balance, furosemide, cumulative dose of chloride, albumin, red blood cell).

### Definitions

The primary exposure factor was the ratio of MAP to the corresponding peak rate of NEQ within the first day. NEQ was calculated by the following formula (all values were in mcg/kg/min except vasopressin, which was in units/hour): NEQ = norepinephrine + epinephrine + phenylephrine / 10 + dopamine / 100 + metaraminol / 8 + vasopressin*2.5 + angiotensin II*10 [10]. The primary outcome was the requirement for RRT between the 2nd and 30th day. The secondary outcome was defined as sustained renal dysfunction, indicated by the final serum creatinine level ≥ 200% of the baseline within 30 days [18]. Baseline creatinine were evaluated using two approaches. First, if the lowest creatinine during this admission fell within the normal range (< 97.2 µmol/L), it was considered the baseline. If no creatinine data were available during entry, we used the simplified Modification of Diet in Renal Disease(MDRD) formula to estimate the baseline. Creatinine (mg/dL) = [75/186/Age^ (-0.203)/ (0.742Female)] ^ (1/-1.154), excluding individuals with CKD [19]. Liquid balance was defined as the total amount of input liquid minus the total amount of output liquid. AKI was defined according to the Kidney Disease: Improving Global Outcomes (KDIGO) criteria [20].

### Sensitivity analyses

First, we reevaluated the relationship between the MAP/NEQ index and the necessity for RRT by substituting the baseline creatinine with AKI. Second, we included both MAP and NEQ, respectively, to gain a more comprehensive understanding of their contributions to RRT. Third, we recalculated the minimum MAP to maximum NEQ ratio, considering that the minimum MAP did not precisely align with the maximum NEQ. Fourth, only patients who received norepinephrine were included. Finally, we excluded the patients with missing MAP corresponding to the maximum NEQ**.**

### Statistical analysis

Variables exhibiting non-normal distribution were presented as medians (interquartile range [IQR]) and compared using the Wilcoxon ranksum test. Categorical variables were described as percentages and compared using the chi-square test. The restricted cubic spline curve explored the nonlinear relationship between the MAP/NEQ index and the requirement for RRT. *Variables with p-values of less than 0.05 were included, and hierarchical backward selection was performed with the Wald test and a significance level of 0.2. In addition, if one variable was eliminated from the model that included all pertinent variables and had more than 10% effect on the target variable, the variable was retained. Then, the variables were selected using the least absolute shrinkage and selection operator (LASSO) regression. The latter two methods were used to check whether the variables selected from the hierarchical backward selection were not missing. The propensity score matching (PSM) was conducted by using the nearest-neighbor matching algorithm with a ratio of 1:2 for RRT(1:1 for sustained renal dysfunction) and a caliper value of 0.02 based on variables with p-values less than 0.05 in the univariate analysis.* Receiver operating characteristic (ROC) curves were plotted to evaluate diagnostic performance. The significance of the variable was assessed using an advanced machine learning algorithm, extreme gradient boosting (XGBoost), which was calculated based on how often each feature was available to split the data. For the missing MAP, an adjacent MAP was used as a substitute. Mean substitution was applied for variables with missing values below 5%. Extreme values were handled by winsorization. Statistical analysis was conducted by R 4.2.3(R Foundation for Statistical Computing, Vienna, Austria), SPSS version 26 (IBM, Armonk, NY, USA), and STATA version 16 (Stata Corp LLC, College Station, Texas 77,845 USA).

## Results

### Baseline characteristics

A total of 5,775 patients were involved, of which 301(5.2%) received RRT and 705 (12.2%) developed sustained renal dysfunction [177 (3.1%) with RRT and 528(9.1%) without RRT] (Supplementary: Table S1). 186 (61.8%) of the RRT group were male with a median age of 62 (IQR: 52–70) years and a median weight of 90 (IQR: 76–110) kg (Table 1). In the sustained renal dysfunction group, the median age and weight were 54 (IQR: 43–71) years and 85 (IQR: 71–102) kg, respectively, and 418 (59.3%) were male (Supplementary Table S2). The median time to RRT initiation was 2.4 (IQR: 1.7–4.4) days; the median time to the last serum creatinine measurement was 6.8 (IQR: 4.4–12.7) days.

**Table 1:** Baseline characteristics and care processes categorized by RRT

Characteristic	Before weighting				After weighting			
	No RRT (n = 5474)	RRT (n = 301)	*P*	SMD	No RRT (n = 464)	RRT (n = 260)	*P*	SMD
Age, years	64 (56–71)	62 (52–70)	0.001	− 0.178	62 (53–71)	62 (53–70)	0.876	0.026
Male	3491 (63.8%)	186 (61.8%)	0.487	− 0.041	288 (62.1%)	157 (60.4%)	0.655	− 0.060
Weight, kg	83 (71–99)	90 (76–110)	< 0.001	0.269	88 (74–104)	88 (75–107)	0.660	− 0.002
Baseline creatinine, µmol/L	61.9 (44.2–70.7)	72.9 (61.9–92.4)	< 0.001	0.612	70.7 (53.0–88.4)	70.7 (61.9–91.0)	0.233	0.003
Hypertension ^a^	2936 (53.6%)	140 (46.5%)	0.016	− 0.143	204 (44.0%)	126 (48.5%)	0.244	0.089
Liver disease	786 (14.4%)	130 (43.2%)	< 0.001	0.582	178 (38.4%)	100 (38.5%)	0.979	− 0.058
*Sepsis shock*	*1208 (22.1%)*	*162 (53.8%)*	< *0.001*	*0.637*	*236 (50.9%)*	*137 (52.7%)*	*0.636*	*− 0.004*
*Cardiac shock*	*376 (6.8%)*	*47 (15.6%)*	< *0.001*	*0.241*	*66 (14.2%)*	*40 (15.4%)*	*0.672*	*0.053*
*Hemorrhagic shock*	*43 (0.8%)*	*3 (1.0%)*	*0.688*	*0.021*	*8 (1.7%)*	*3 (1.2%)*	*0.547*	*− 0.058*
*Obstructive shock*^*b*^	*186 (3.4%)*	*19 (6.3%)*	< *0.001*	*0.120*	*31 (6.7%)*	*17 (6.5%)*	*0.942*	*0.000*
*Unspecified shock*	*3661 (66.9%)*	*70 (23.3%)*	< *0.001*	*0.135*	*123 (26.5%)*	*63 (24.2%)*	*0.501*	*− 0.082*
Duration of MAP < 65 mmHg, h			< 0.001	0.266			0.824	− 0.068
< 5	3716 (67.9%)	167 (55.4%)			264 (56.9%)	154 (59.2%)		
5–10	1130 (20.6%)	73 (24.3%)			109 (23.5%)	57 (21.9%)		
> 10	628 (11.5%)	61 (20.3%)			91 (19.6%)	49 (18.9%)		
Fluid Balance, L	1.79 (0.44–3.52)	4.64 (2.17–8.75)	< 0.001	0.697	3.86 (1.64–6.84)	4.25 (1.76–8.05)	0.205	0.009
Highest chloride level, mmol/L			< 0.001	− 0.306			0.664	− 0.067
< 108	2412 (44.1%)	168 (55.8%)			250 (53.9%)	143 (55.0%)		
108–111	1752 (32.0%)	72 (23.9%)			112 (24.1%)	67 (25.8%)		
> 111	1310 (23.9%)	61 (20.3%)			102 (22.0%)	50 (19.2%)		
Inotropic support ^c^	353 (6.5%)	47 (15.6%)	< 0.001	0.253	64 (13.8%)	38 (14.6%)	0.760	0.016
Vancomycin administration	3201 (58.5%)	221 (73.4%)	< 0.001	0.338	327 (70.5%)	188 (72.3%)	0.601	0.022
Red blood cell transfusion	1411 (25.8)	115 (38.2%)	< 0.001	0.256	171 (36.9%)	91 (35.0%)	0.619	− 0.044
Invasive mechanical ventilation	3985 (72.8%)	265 (88.0%)	< 0.001	0.470	389 (83.8%)	226 (86.9%)	0.265	0.059
Highest lactate level > 2 mmol/L	3767 (68.8%)	259 (86.1%)	< 0.001	0.497	390 (84.1%)	218 (83.9%)	0.942	− 0.050
Lowest platelet count, × 10^9^/L			< 0.001	0.258			0.818	− 0.007
> 150	2643 (48.3%)	98 (32.6%)			171 (36.8%)	96 (36.9%)		
100–150	1725 (31.5%)	72 (23.9%)			116 (25.0%)	60 (23.1%)		
< 100	1106 (20.2%)	131 (43.5%)			177 (38.2%)	104 (40.0%)		
MAP/NEQ, mmHg/mcg/kg/min	385.4 (159.7–665.9)	17.2 (9.2–268.1)	< 0.001		153.5 (10.9–402.9)	40.9 (9.6–284.7)	< 0.001	
SOFA	7 (5–9)	12 (10–14)	< 0.001		9 (8–12)	11 (9–14)	< 0.001	
APS III	44 (33–62)	80 (66–97)	< 0.001		61 (47–78)	79 (63–94)	< 0.001	
Highest NEQ, mcg/kg/min			< 0.001				0.008	
< 0.10	1056 (19.3%)	18 (6.0%)			48 (10.3%)	17 (6.5%)		
0.10–0.20	1892 (34.6%)	34 (11.3%)			86 (18.6%)	31 (11.9%)		
> 0.20	2526 (46.1%)	249 (82.7%)			330 (71.1%)	212 (81.6%)		
Chronic pulmonary disease	1418 (25.9%)	89 (29.6%)	0.159		122 (26.3%)	28 (30.0%)	0.285	
Congestive heart failure	1528 (27.9%)	99 (32.9%)	0.062		149 (32.1%)	87 (33.5%)	0.710	
Diabetes mellitus	1510 (27.6%)	88 (29.2%)	0.533		135 (29.1%)	74 (28.5%)	0.857	
Admission type			< 0.001				0.176	
Elective	1883 (34.4%)	61 (20.3%)			102 (22.0%)	54 (20.8%)		
Emergency	2304 (42.1%)	146 (48.5%)			247 (53.2%)	125 (48.1%)		
Urgent	1287 (23.5%)	94 (31.2%)			115 (24.8%)	81 (31.1%)		
MAP^d^, mmHg	67 (60–74)	65 (58–73)	0.012		66 (59–73)	65 (58–74)	0.738	
MAP^e^, mmHg	56 (50–60)	52 (43–58)	< 0.001		53 (46–58)	53 (44–58)	0.348	
Albumin transfusion	2291 (41.9%)	127 (42.2%)	0.907		190 (41.0%)	105 (40.4%)	0.882	
Furosemide administration	1486 (27.2%)	76 (25.3%)	0.471		113 (24.4%)	69 (26.5%)	0.516	
Cumulative chloride dose, mmol			< 0.001				0.577	
< 500	3177 (58.0%)	149 (49.5%)			216 (46.6%)	131 (50.4%)		
500–1000	1881 (34.4%)	94 (31.2%)			157 (33.8%)	84 (32.3%)		
> 1000	416 (7.6%)	58 (19.3%)			91 (19.6%)	45 (17.3%)		

Values were median (IQR) or n (%)

*RRT* renal replacement therapy, *SMD* standardized mean difference, *MAP* mean arterial pressure, *NEQ* norepinephrine equivalent dose, *SOFA* Sequential Organ Failure Assessment, *APS III* Acute Physiology Score III

^a^Diagnosis based on the recorded ICD-9 and ICD-10 codes [21]

^b^Obstructive shock mainly included cardiac tamponade, pulmonary embolism, tension pneumothorax, and aortic dissection

^c^Inotropic support was defined as the administration of dobutamine or milrinone

^d^MAP corresponding to the peak rate of vasopressor

^e^Lowest MAP within 24 hours after ICU admission

The median IQR for the MAP/NEQ index was markedly lower in the RRT group, at 17.2 (IQR: 9.2–268.1) mmHg/mcg/kg/min, compared to 385.4 (IQR: 159.7–665.9) mmHg/mcg/kg/min in the No RRT group (*P* < 0.001). The MAP/NEQ index remained statistically different after PSM (Table 1). Similar results could be found in the sustained renal dysfunction group (Supplementary: Table S2). Considering the maximum and average rates, norepinephrine was the highest in both groups, with a more significant difference in the RRT group than in the No RRT group (Supplementary: Table S3). In both groups, norepinephrine accounted for over half of all vasopressors (Supplementary: Table S4). AKI was prevalent among patients receiving vasopressors, with the maximum injury typically occurring within three days. Stage 3 was more frequent in the RRT group, whereas stages 1 and 2 were more common in the No RRT group (Supplementary: Table S5).

### Primary outcome

**Fig. 2:**
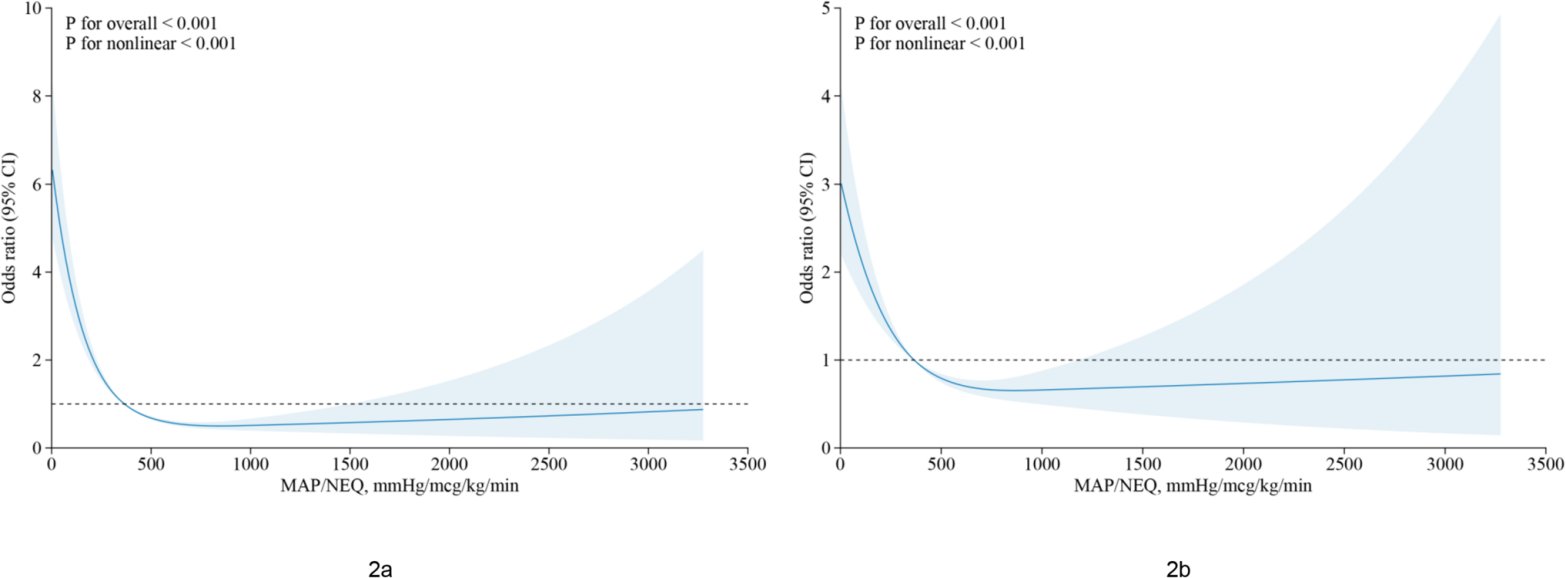
Unadjusted (**a**) and adjusted (**b**) restricted cubic spline curves showing OR and 95% CI for the risk of RRT by the MAP/NEQ index*OR* odds ratio, *CI* confidence intervals, *RRT* renal replacement therapy, *MAP* mean arterial pressure, *NEQ* norepinephrine equivalent dose

**Fig. 3:**
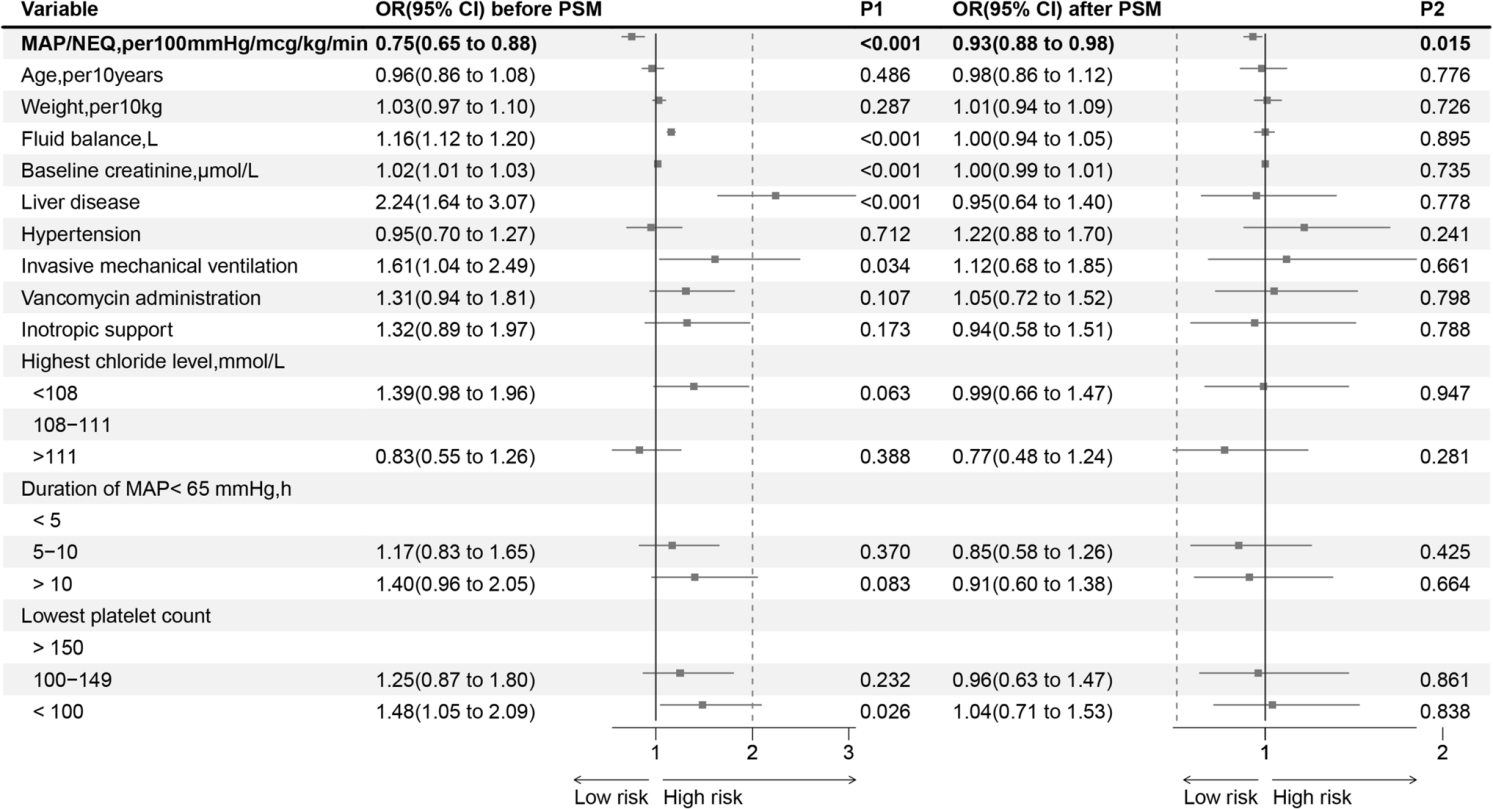
Forest plot of risk factors for RRT within 30 days using multivariable logistic regression analysis. *OR* odds ratio, *CI* confidence interval, *PSM* propensity score matching, *P1* *P* value before propensity score matching, *P2* *P* value after propensity score matching, *MAP* mean arterial pressure, *NEQ* norepinephrine equivalent dose, *RRT* renal replacement therapy, Inotropic support was defined as the administration of dobutamine or milrinone

**Fig. 4:**
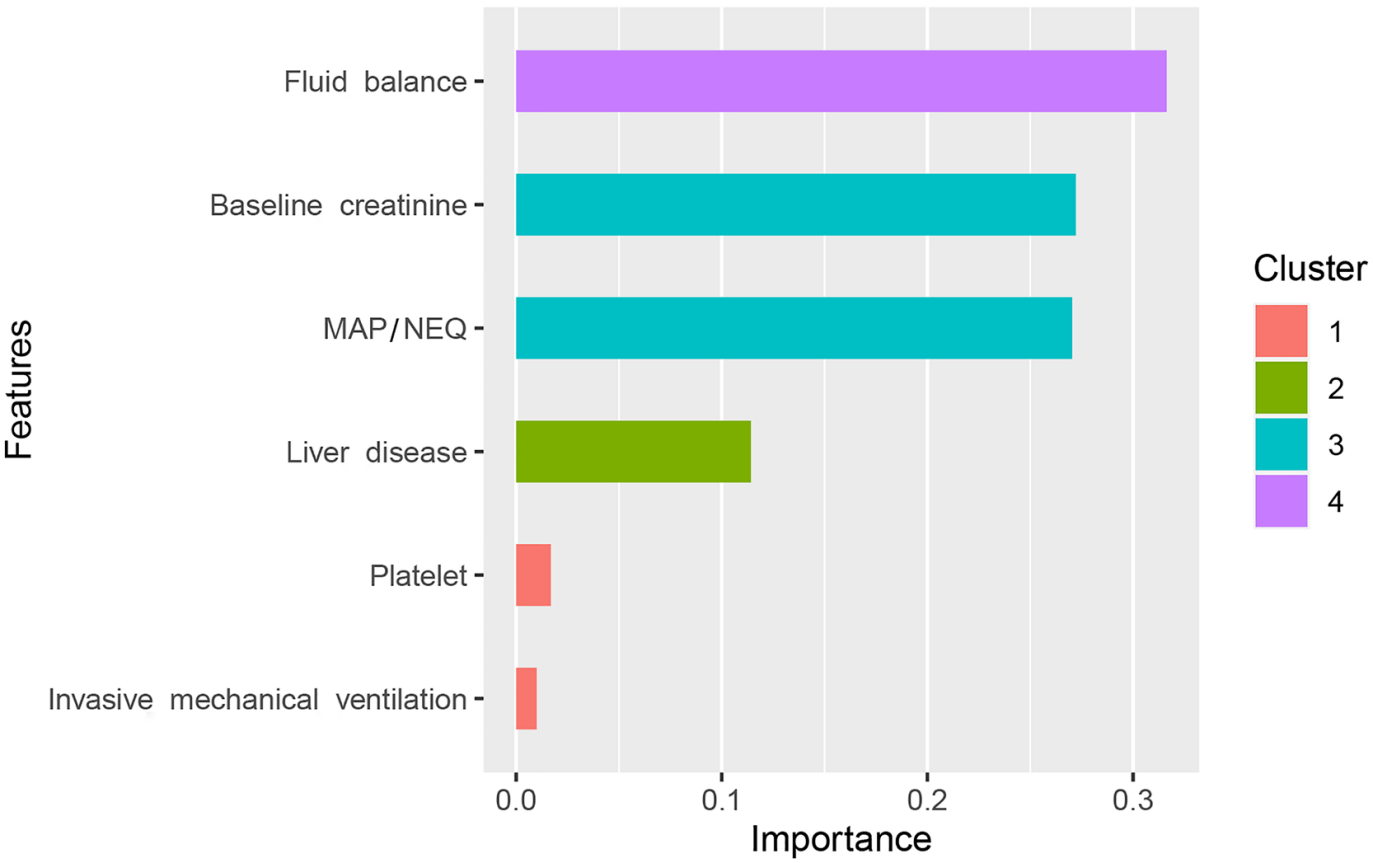
The relative importance of variables associated with RRT by machine learning methods using XGBoost within 30 days. *MAP* mean arterial pressure, *NEQ* norepinephrine equivalent dose, *RRT* renal replacement therapy, *XGBoost* extreme gradient boosting

In the univariate logistic regression analysis, 24 characteristics (including dummy variables) were associated with the outcome (Supplementary: Table S6) and the variables screened by LASSO regression were presented in the Supplementary: Fig.S1. Unadjusted (Fig. 2a) and adjusted (Fig. 2b) restricted cubic spline analyses revealed a discernible decreasing L-shaped relationship between the MAP/NEQ index and the requirement for RRT. For each increase of 100 mmHg/mcg/kg/min, the adjusted odds ratio (OR) was 0.75 (95% CI 0.65–0.88, *P* < 0.001) when the MAP/NEQ index was ≤ 365.5 mmHg/mcg/kg/min before PSM and 0.93 (95% CI 0.88–0.98, *P* = 0.015) after PSM. In addition, fluid balance, baseline creatinine, MAP/NEQ index, liver disease, invasive mechanical ventilation, and platelet were independently associated with an increased risk of RRT (Fig. 3). The adjusted OR was 0.77(95% CI 0.66–0.89, *P* = 0.001) for every 100 mmHg/mcg/kg/min increase when the MAP/NEQ index was ≤ 365.5 mmHg/mcg/kg/min adjusted for early AKI before PSM and 0.93(95% CI 0.87–0.98, *P* = 0.017) after PSM (Supplementary: Fig.S2 model1). The adjusted OR for every 5 mmHg increase in MAP and every 0.01 mcg/kg/min increase in NEQ for the MAP/NEQ index ≤ 365.5 mmHg/mcg/kg/min were 0.99 (95% CI 0.98–1.01, *P* = 0.708) and 1.10 (95% CI 1.05–1.15, *P* < 0.001), respectively, showing general consistency after PSM (Supplementary: Fig.S2 model2). When adjusted for the lowest MAP rather than the MAP corresponding to the maximum NEQ, the adjusted OR per 100 mmHg/mcg/kg/min increase was 0.74 (95% CI 0.61–0.91, *P* = 0.005) for the MAP/NEQ index ≤ 290.3 mmHg/mcg/kg/min before PSM. After PSM, the adjusted OR was 0.92 (95% CI 0.86–0.98, *P* = 0.017) (Supplementary: Fig.S2 model3). When including only patients who received norepinephrine, the adjusted OR per 100 mmHg/mcg/kg/min increase was 0.65 (95% CI 0.46–0.91, *P* = 0.015) for the MAP/NEQ index ≤ 227.2 mmHg/mcg/kg/min before PSM (Supplementary: Fig.S2 model4). The OR increased slightly but was still statistically significant after PSM (OR: 0.91, 95% CI 0.86–0.97, *P* = 0.006). After excluding patients with missing MAP corresponding to maximum NEQ, the OR was similar to that without removing missing MAP (Supplementary: Fig.S2 model5). The top three variables’ relative importance were fluid balance, baseline creatinine, and the MAP/NEQ index (Fig. 4).

The threshold for MAP to predict the need for RRT was 64 mmHg (specificity, 45.2%; sensitivity, 62.9%), with an area under the ROC curve (AUC) of 54.3% (95% CI 50.8–57.8; *P* = 0.012, Fig.S3a) and 0.41 mcg/kg/min of NEQ (specificity, 75.6%; sensitivity, 64.1%), with an AUC of 75.8% (95% CI, 72.9–78.8; *P* < 0.001, Fig.S3b). The threshold of the MAP/NEQ index to predict RRT was 161.7 mmHg/mcg/kg/min (specificity, 65.8%; sensitivity, 74.8%), with an AUC of 75.9% (95% CI, 73.1–78.8; *P* < 0.001, Fig.S3c). The discriminatory capacity of the MAP/NEQ index for predicting RRT was significantly superior to that of MAP (*P* < 0.001) but not different from that of NEQ (*P* = 0.967); however, the MAP/NEQ index additionally identified 3.1% of patients with hypertension and 5.1% of patients without hypertension for the requirement of RRT based on the NEQ without actually receiving RRT. In addition, 7.6% of patients with hypertension and 8.9% of patients without hypertension who were judged not to require RRT according to the NEQ threshold but actually received RRT were able to be recognized by the MAP/NEQ index.

### Secondary outcome

In the multivariate logistic regression, an increase of each 100 mmHg/mcg/kg/min increase led to an adjusted OR of 0.84 (95% CI 0.76–0.93;* P* = 0.001) for sustained renal dysfunction, below the cutoff of 365.7 mmHg/mcg/kg/min before PSM and 0.94 (95% CI 0.90–0.97; *P* = 0.001) after PSM (Supplementary: Fig.S4). Forward selection logistic regression analyses showed the consistency between the MAP/NEQ index and sustained renal dysfunction (Supplementary: Table S7). For predicting sustained renal dysfunction, the MAP threshold was 63 mmHg (specificity: 41.6%, sensitivity: 67.3%) with an AUC of 54.7% (95% CI 52.3–57.0; *P* < 0.001, Supplementary: Fig.S5a). The threshold of NEQ was 0.21 mcg/kg/min (specificity: 62.8%, sensitivity: 63.5%) with an AUC of 67.3% (95% CI 65.2–69.4; *P* < 0.001, Supplementary: Fig.S5b). The threshold of the MAP/NEQ index was 309.9 mmHg/mcg/kg/min (specificity: 66.1%, sensitivity: 61.7%) with an AUC of 67.7% (95% CI 65.6–69.8; *P* < 0.001, Supplementary: Fig.S5c). The discriminatory capacity of the MAP/NEQ index for predicting sustained renal dysfunction was significantly superior to that of MAP (*P* < 0.001) but not statistically different from that of the NEQ (*P* = 0.791).

## Discussion

### Key findings

First, patients with hemodynamic instability exhibited a significant prevalence of AKI. A notable association between the MAP/NEQ index and RRT showed a declining L-shaped relationship. When examining the relative importance, fluid balance, baseline creatinine, and the MAP/NEQ index stood out as the pivotal factors. Second, The MAP/NEQ index ≤ 161.7 mmHg/mcg/kg/min was recommended to evaluate the initiation of RRT for patients who received at least one vasopressor over 6 h within the first 24 h of ICU admission. The final discovery was that dynamic adjustment of the volume-oriented MAP/NEQ index could potentially be a new strategy for individualized clinical treatment applications.

### Relationship with previous studies

Our findings partially contribute to further calculating the MAP/NEQ index, building upon previous studies that have quantified the correlation between MAP, various vasopressors, and RRT. Determining the optimal timing for initiating RRT is challenging in the clinical setting. This underscores the need for further research and more nuanced analysis methods, such as the MAP/NEQ index, to manage such cases better.

The requirement for RRT does not correlate precisely with MAP. The possible reasons are that the occurrence of AKI involves intricate mechanisms such as the regulation of renal afferent and efferent arterioles, uneven distribution of corticomedullary blood flow, endothelial dysfunction, intravascular microthrombosis, and glycocalyx damage [9]. These factors account for the potential variations in the MAP required for different disease types and stages of the same disease. For instance, some studies have indicated that MAP is not a risk factor for AKI but MPP, representing the actual organ perfusion pressure. However, prehospital minimum systolic blood pressure or minimum prehospital MAP is an independent risk factor for predicting AKI in trauma patients [22, 23]. In addition, intraoperative hypotension is associated with persistent nephropathy as opposed to delayed nephropathy [24]. These findings suggest that the role of individualized MAP in different diseases complicated by AKI still requires further exploration.

Half of the vasopressors were norepinephrine, which aligns with the guideline recommendation [25]. Mechanistically, critically ill patients often exhibit a relative deficiency in catecholamine and receptor downregulation. A step-down treatment strategy, similar to broad-spectrum antibiotics for septic shock, has been proposed, wherein multiple vasopressors are combined in small doses and subsequently tapered [26]. Previous studies have examined the association of vasopressors with AKI [27–29]. Therefore, it is crucial to quantify and optimize the vasopressor dose. The widely used formula proposed by Goradia et al. in 2020 has gained prominence [10].

For the diagnosis of AKI and the requirement for RRT, urine output and creatinine have the disadvantage of low specificity and delay, respectively. At the same time, biomarkers such as plasma neutrophil gelatinase-associated lipocalin (NGAL) and electronic risk algorithms have relative shortcomings, such as invasive test performance, high cost, variable sensitivity and specificity, and reliance on large amounts of data, which are not widely applied in clinical practice[1, 28]. In contrast, The MAP/NEQ index provides early reference guidelines. This study defined the threshold for initiating RRT as 161.7 mmHg/mcg/kg/min. We calculated the percentage of patients with no change in urine output, and the MAP/NEQ index reaching the threshold for RRT was 46.8% among the patients who received RRT. However, the MAP/NEQ index only served as an average predictor of sustained renal dysfunction. Interestingly, this study suggests that using MAP and NEQ to assess the need for RRT might be more effective than relying on either. Moreover, the prioritization of influential factors on the need for RRT, with baseline creatinine ranking subsequent to fluid balance, is not entirely consistent with the study conducted by Mele A et al. We presumably incorporated different inclusion criteria and endpoint indicators [30].

Although vasopressors may somewhat elevate the MAP, they can also exacerbate renal microcirculatory disorders. Concurrently, the adequate perfusion pressure in the kidneys diminishes, leading to a further increase in NEQ. The association between the MAP/NEQ index and the RRT may reflect the degree of reverse causality. Taking into consideration the study by Mele A et al., where cumulative fluid balance in terms of urine volume, rather than fluid input, was linked to RRT, we propose that the combination of individualized MAP (not necessarily 65 mmHg) and NEQ, with dynamic adjustment of the MAP/NEQ index oriented to urine volume, may have the potential to optimize MAP and ameliorate acute renal complications in the ICU [30].

### Strengths and limitations

To our knowledge, this is the inaugural study to employ the MAP/NEQ index in determining RRT requirements. We applied the restricted cubic spline curve to clarify the trend that as the MAP/NEQ index increased, the risk of the need for RRT decreased. Furthermore, we provided estimations of the significance of the exposure variables. Finally, our findings were consistent with the sensitivity analyses and PSM analyses, providing robustness.

Our study had certain limitations. First, this was a single-center retrospective observational study. Second, we did not provide specific details regarding the timing of vasopressor administration. Third, we did not assess changes in exposure variables after the first day in the ICU. Fourth, we lacked data on inflammatory indicators, and some baseline imbalances might have existed. *Fifth, the timing of RRT initiation was unclear, and this decision was left to the attending physician's discretion. Sixth, we did not assess the effect of specific treatment on AKI.* Finally, owing to the utilization of different formulas for converting NEQ, different thresholds were generated.

## Conclusions

The MAP/NEQ index emerged as an alternative metric to NEQ for the necessity of RRT for adult patients who received at least one vasopressor over 6 hours within the first 24 hours of ICU admission. The dynamic regulation of the MAP/NEQ index facilitated by the synergistic utilization of various low-dose vasopressors in conjunction with urine volume orientation may prove beneficial in exploring personalized optimization of MAP.

### Supplementary Information

Below is the link to the electronic supplementary material.

Supplementary file1 (TIF 539 KB)

Supplementary file2 (TIF 522 KB)

Supplementary file3 (TIF 843 KB)

Supplementary file4 (TIF 2556 KB)

Supplementary file5 (TIF 480 KB)

Supplementary file6 (TIF 466 KB)

Supplementary file7 (TIF 477 KB)

Supplementary file8 (TIF 2003 KB)

Supplementary file9 (TIF 478 KB)

Supplementary file10 (TIF 473 KB)

Supplementary file11 (TIF 483 KB)

Supplementary file12 (DOC 3125 KB)

## Data Availability

The datasets are stored in the physionet. (https://physionet.org/content/mimiciv/2.2/).
