# Supplementary figures and images for: Mean arterial pressure to norepinephrine equivalent dose ratio for predicting renal replacement therapy requirement: a retrospective analysis from the MIMIC-IV
Source: Int Urol Nephrol. 2024 Jan 18;56(6):2065–74. doi: 10.1007/s11255-023-03908-3 (PMC11090965; doi:10.1007/s11255-023-03908-3)

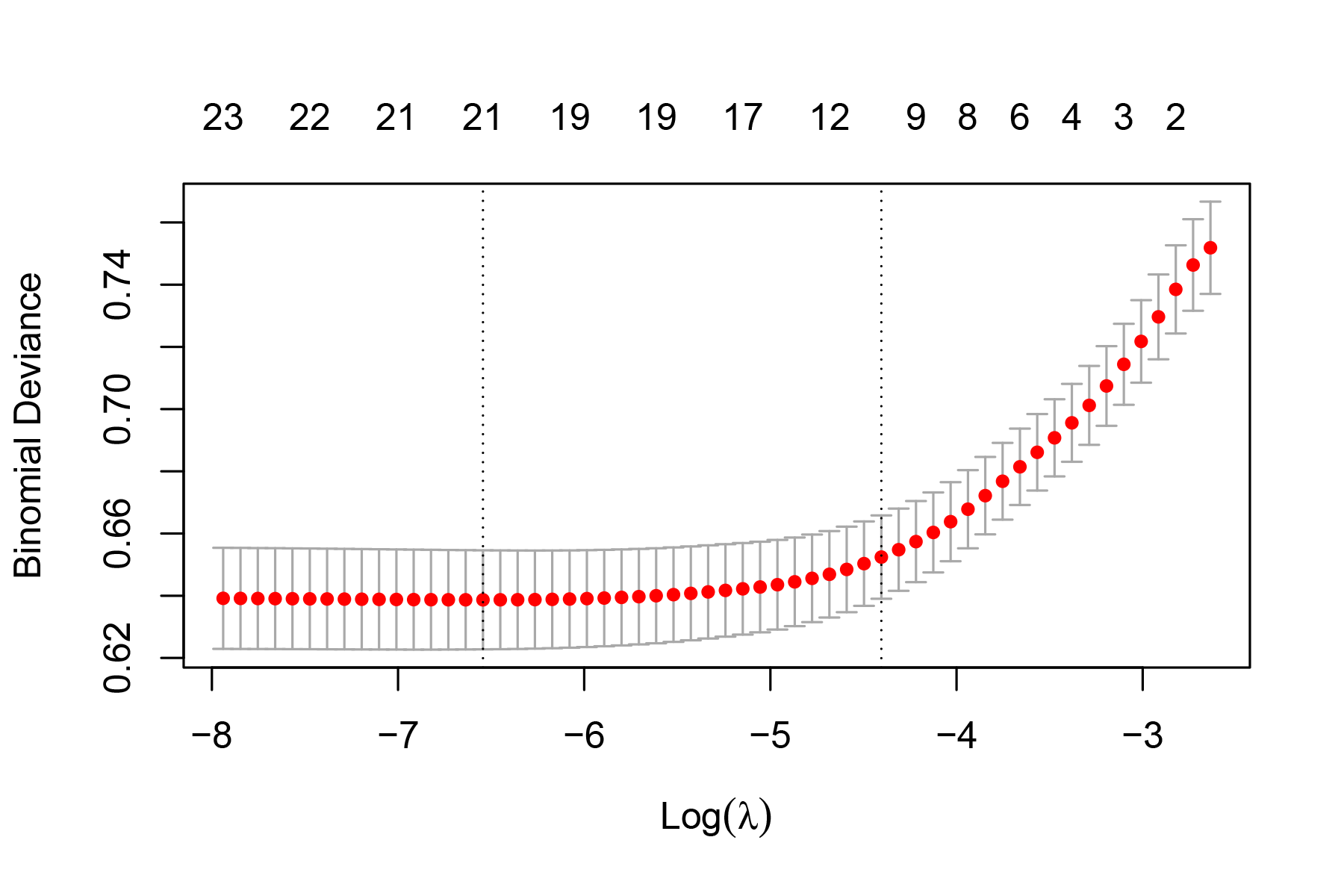

Supplement: Supplementary file 1 — Supplementary file1 (TIF 539 KB) [file 11255_2023_3908_MOESM1_ESM.tif]

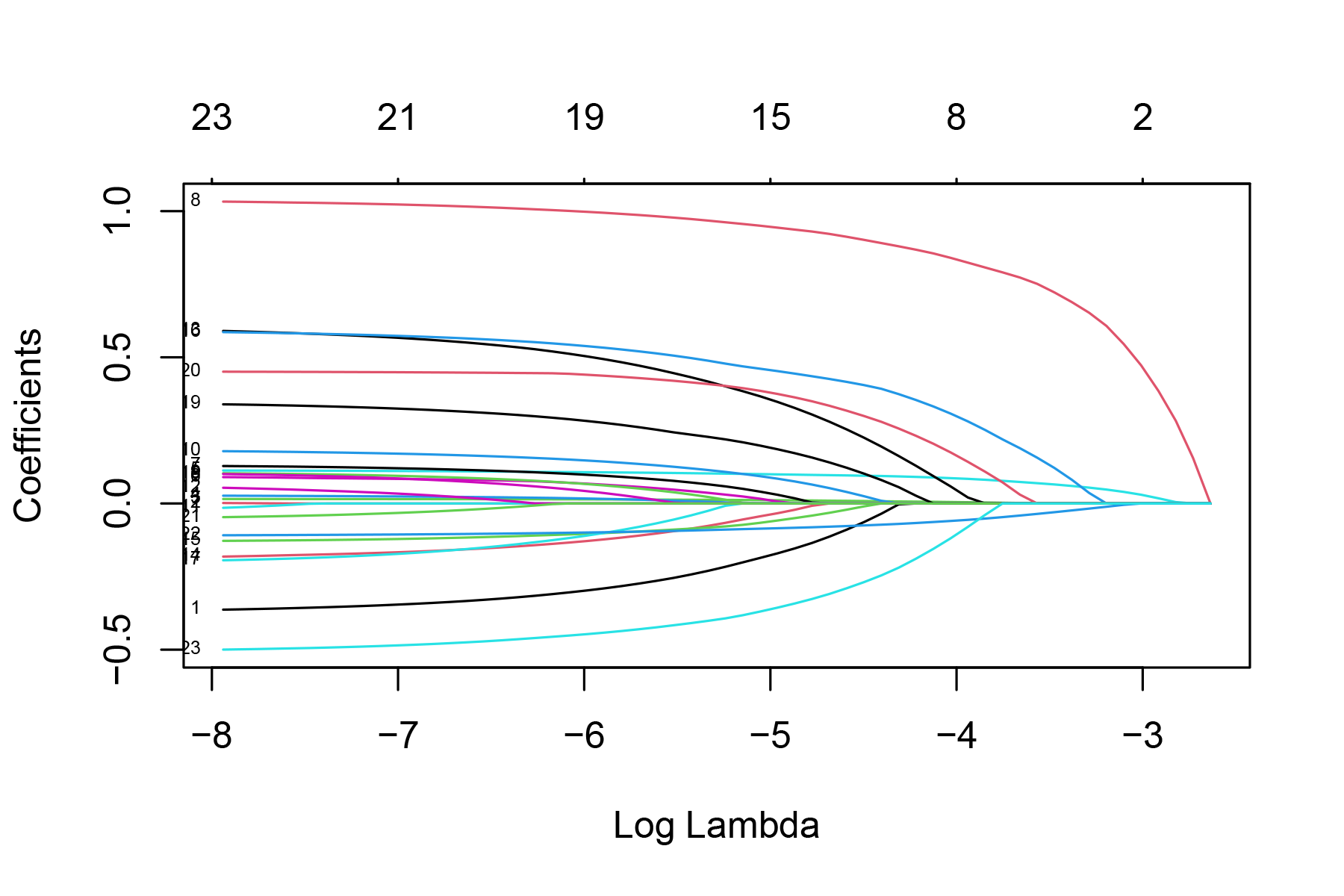

Supplement: Supplementary file 2 — Supplementary file2 (TIF 522 KB) [file 11255_2023_3908_MOESM2_ESM.tif]

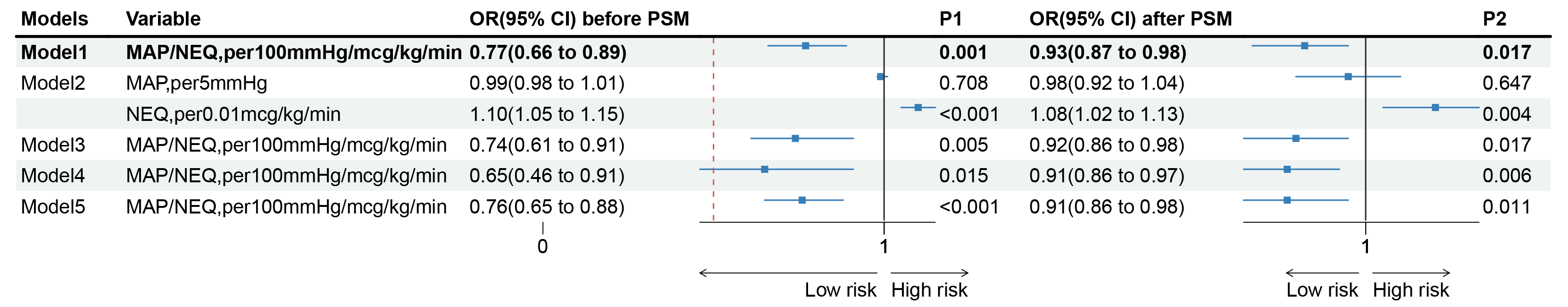

Supplement: Supplementary file 3 — Supplementary file3 (TIF 843 KB) [file 11255_2023_3908_MOESM3_ESM.tif]

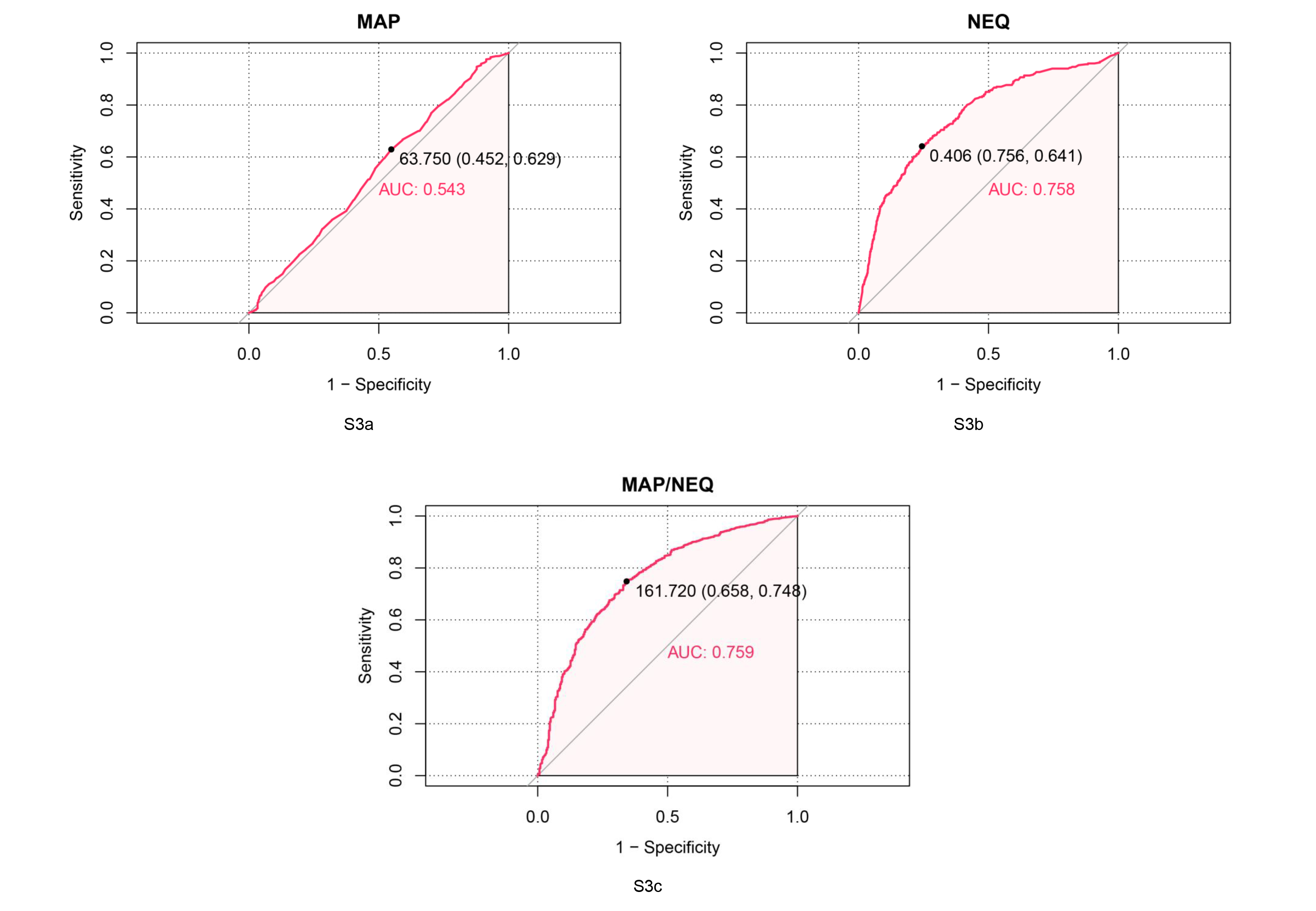

Supplement: Supplementary file 4 — Supplementary file4 (TIF 2556 KB) [file 11255_2023_3908_MOESM4_ESM.tif]

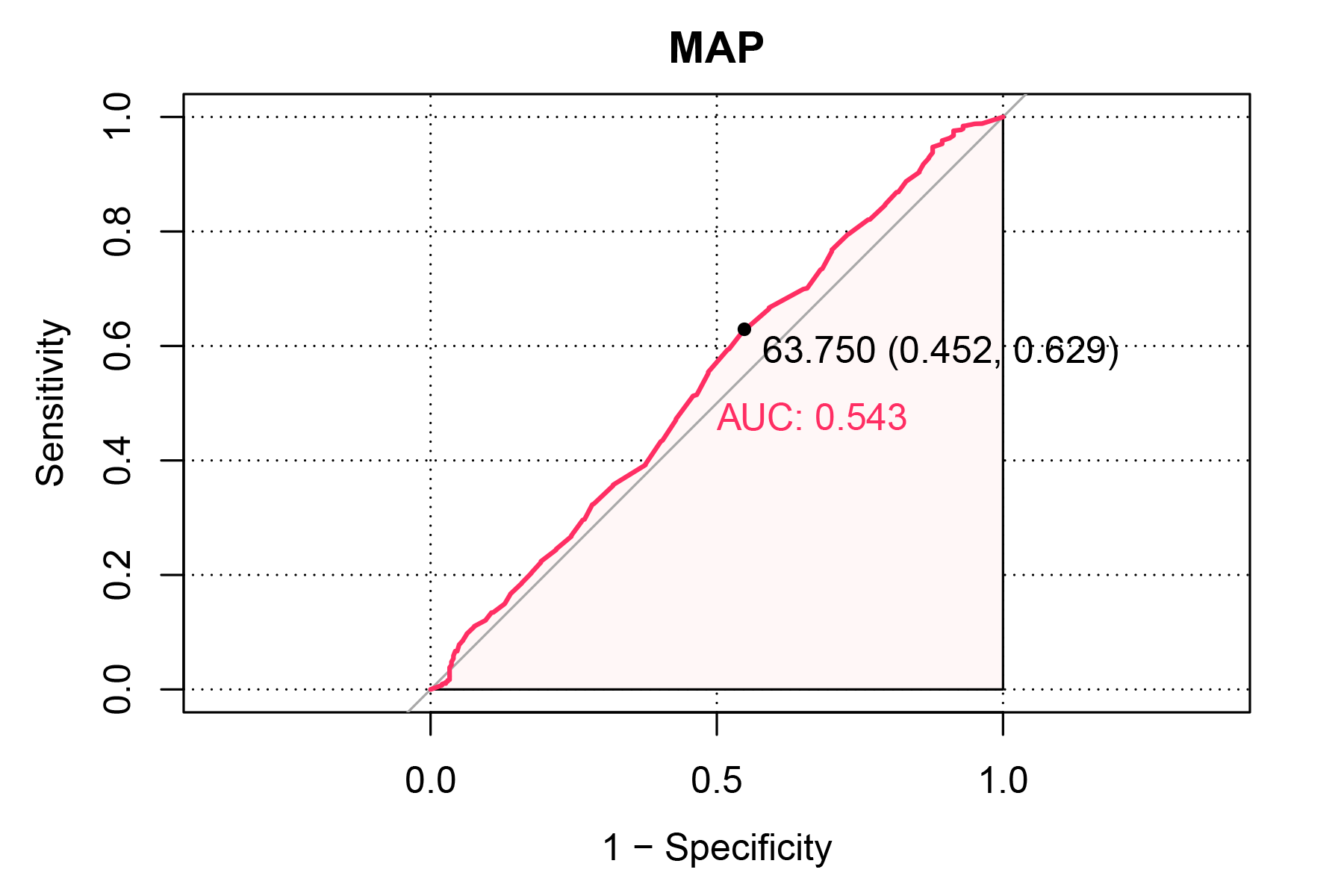

Supplement: Supplementary file 5 — Supplementary file5 (TIF 480 KB) [file 11255_2023_3908_MOESM5_ESM.tif]

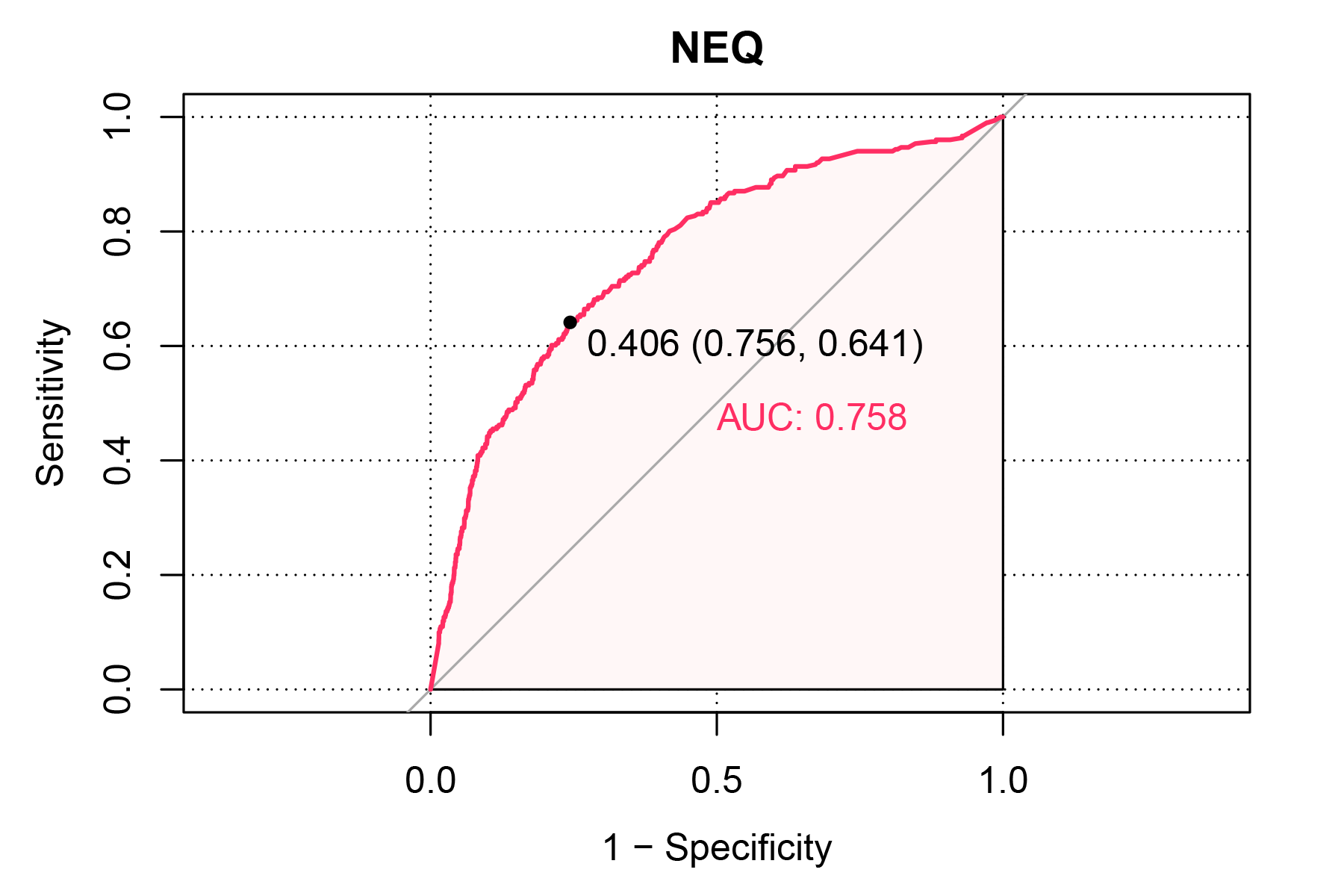

Supplement: Supplementary file 6 — Supplementary file6 (TIF 466 KB) [file 11255_2023_3908_MOESM6_ESM.tif]

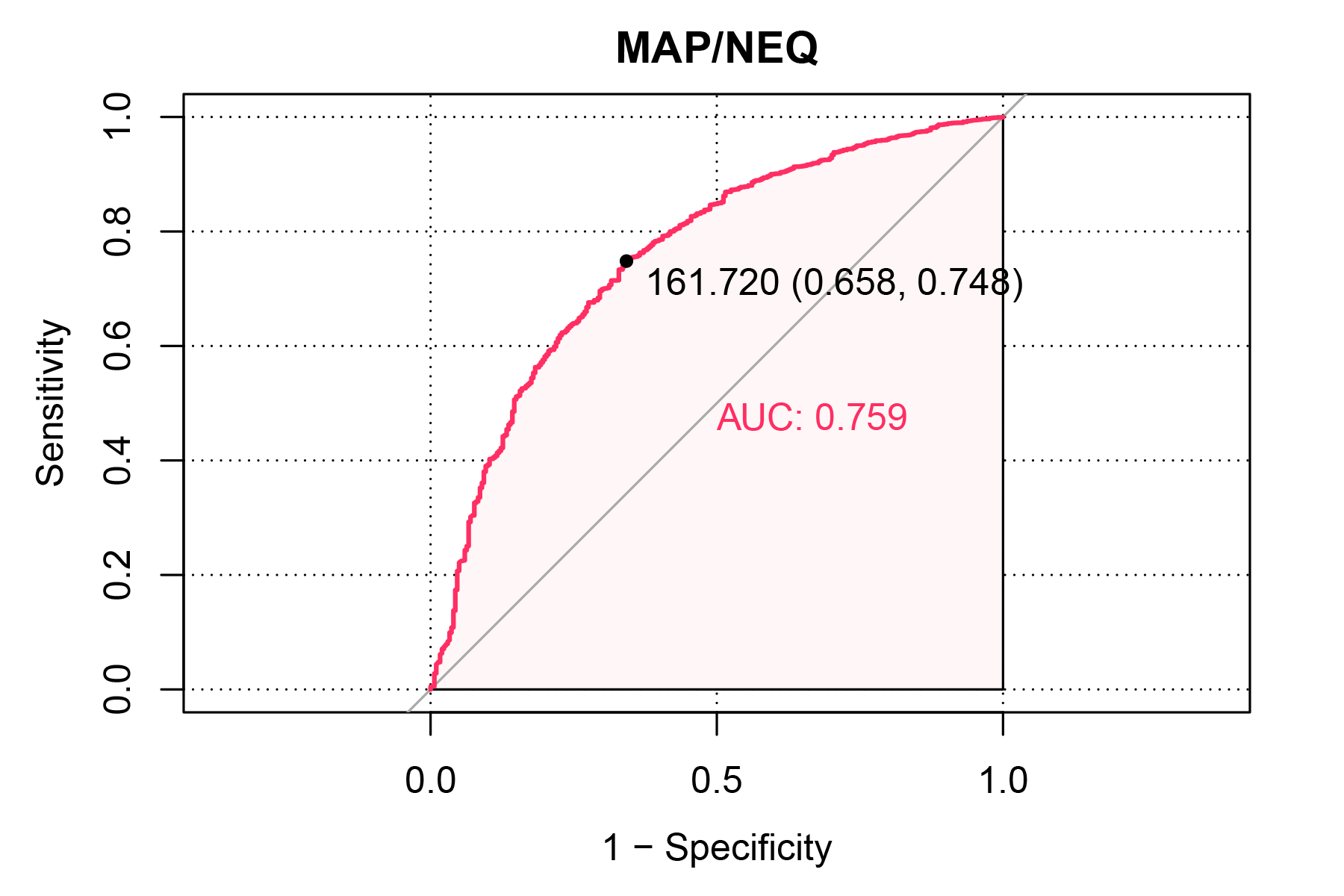

Supplement: Supplementary file 7 — Supplementary file7 (TIF 477 KB) [file 11255_2023_3908_MOESM7_ESM.tif]

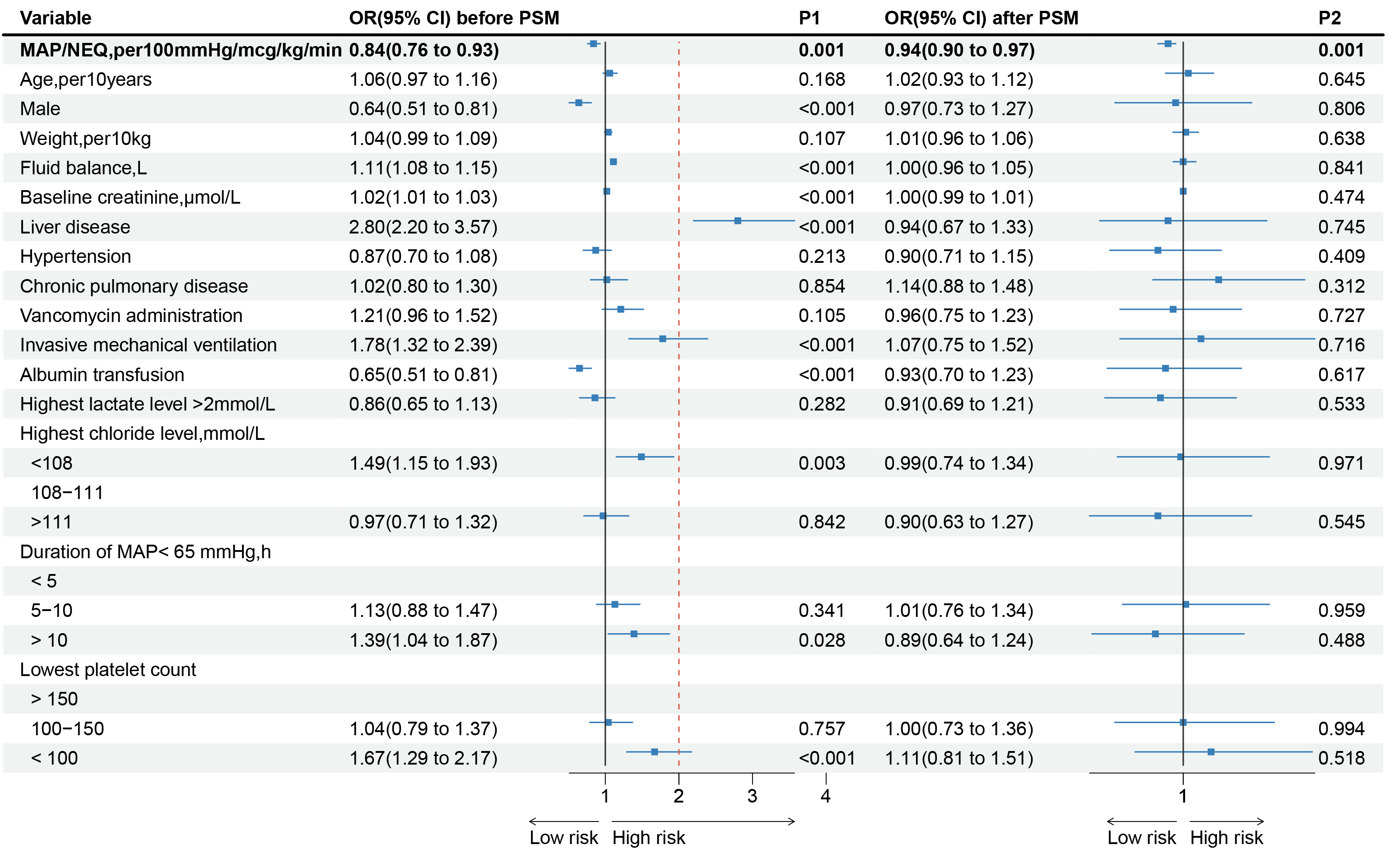

Supplement: Supplementary file 8 — Supplementary file8 (TIF 2003 KB) [file 11255_2023_3908_MOESM8_ESM.tif]

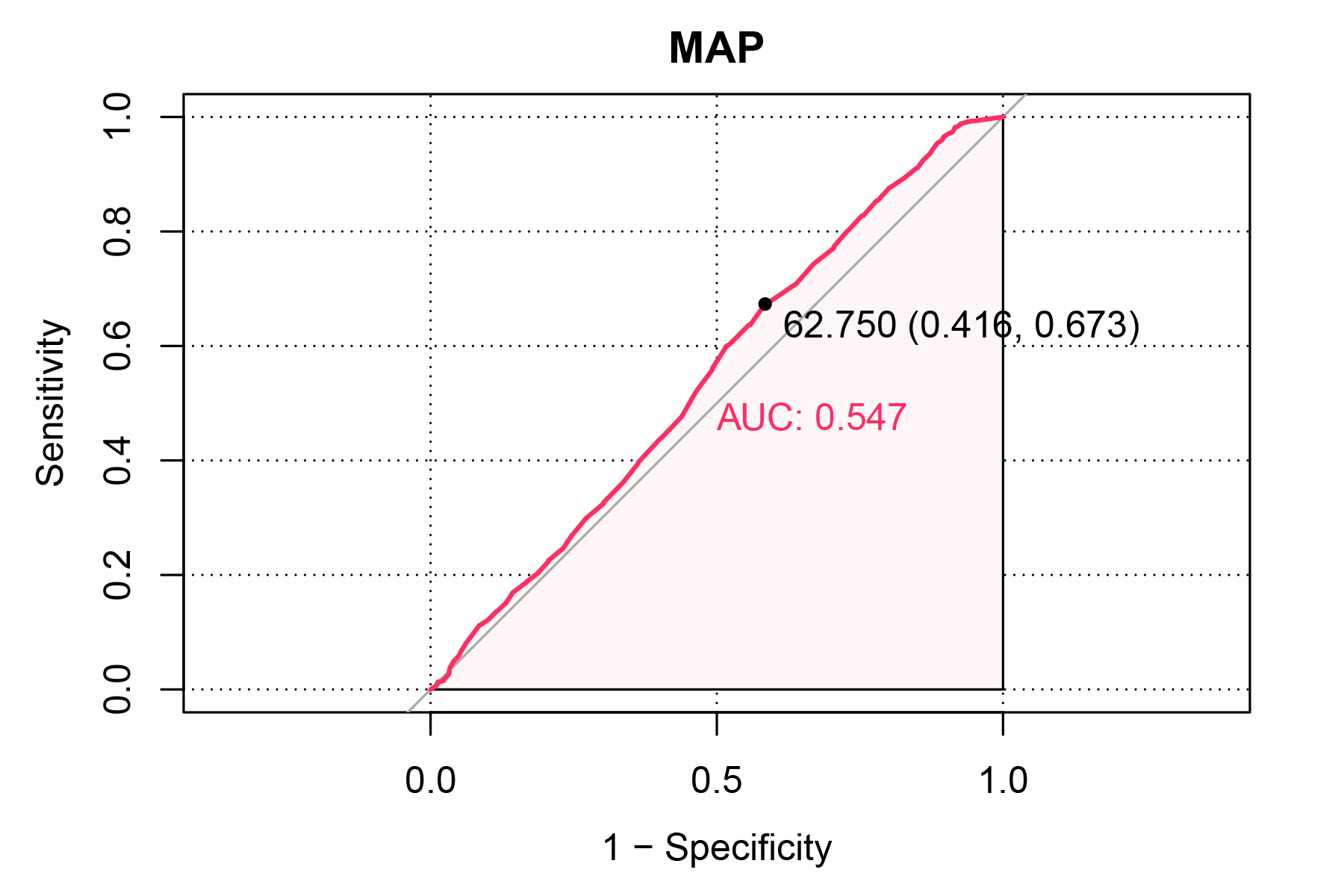

Supplement: Supplementary file 9 — Supplementary file9 (TIF 478 KB) [file 11255_2023_3908_MOESM9_ESM.tif]

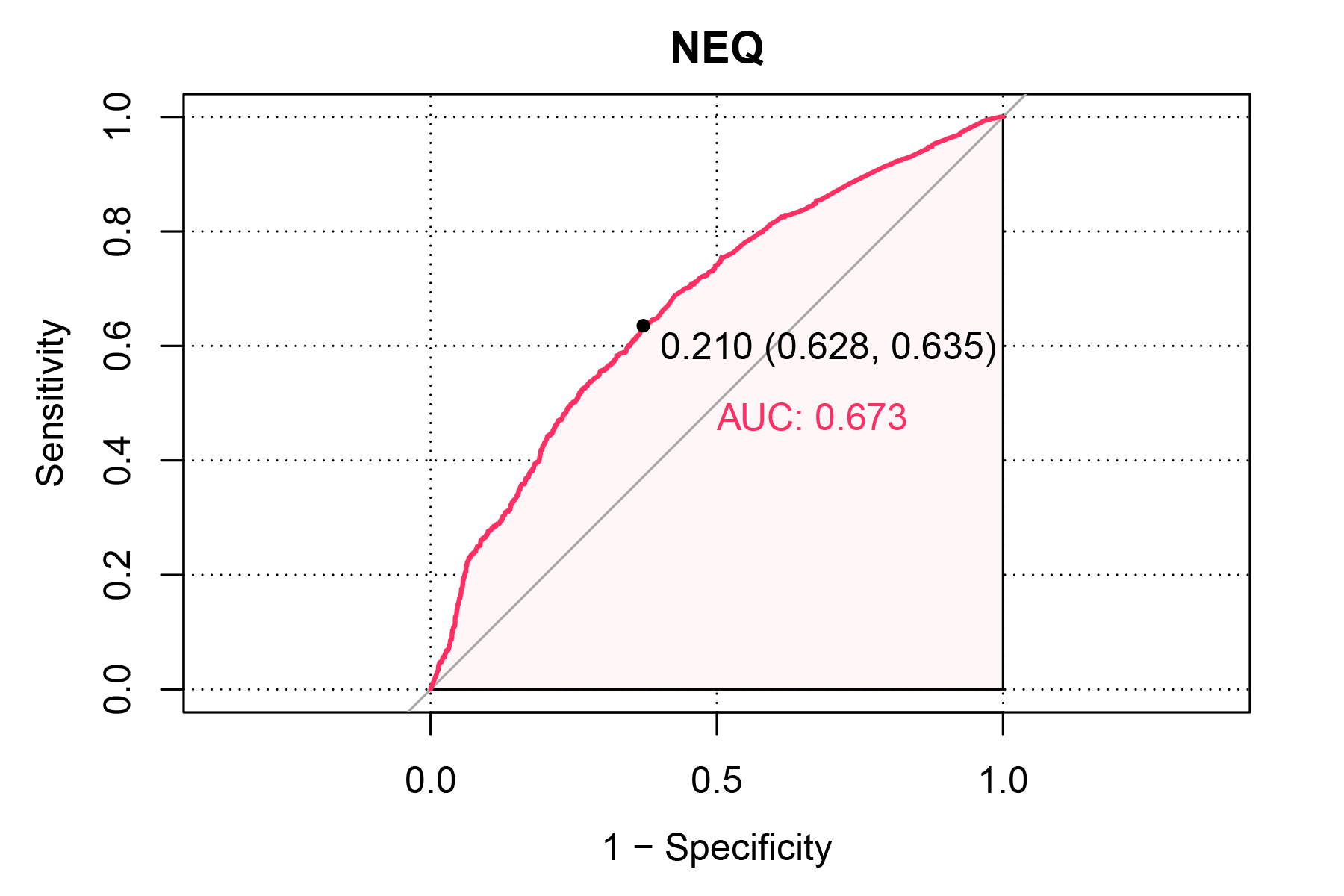

Supplement: Supplementary file 10 — Supplementary file10 (TIF 473 KB) [file 11255_2023_3908_MOESM10_ESM.tif]

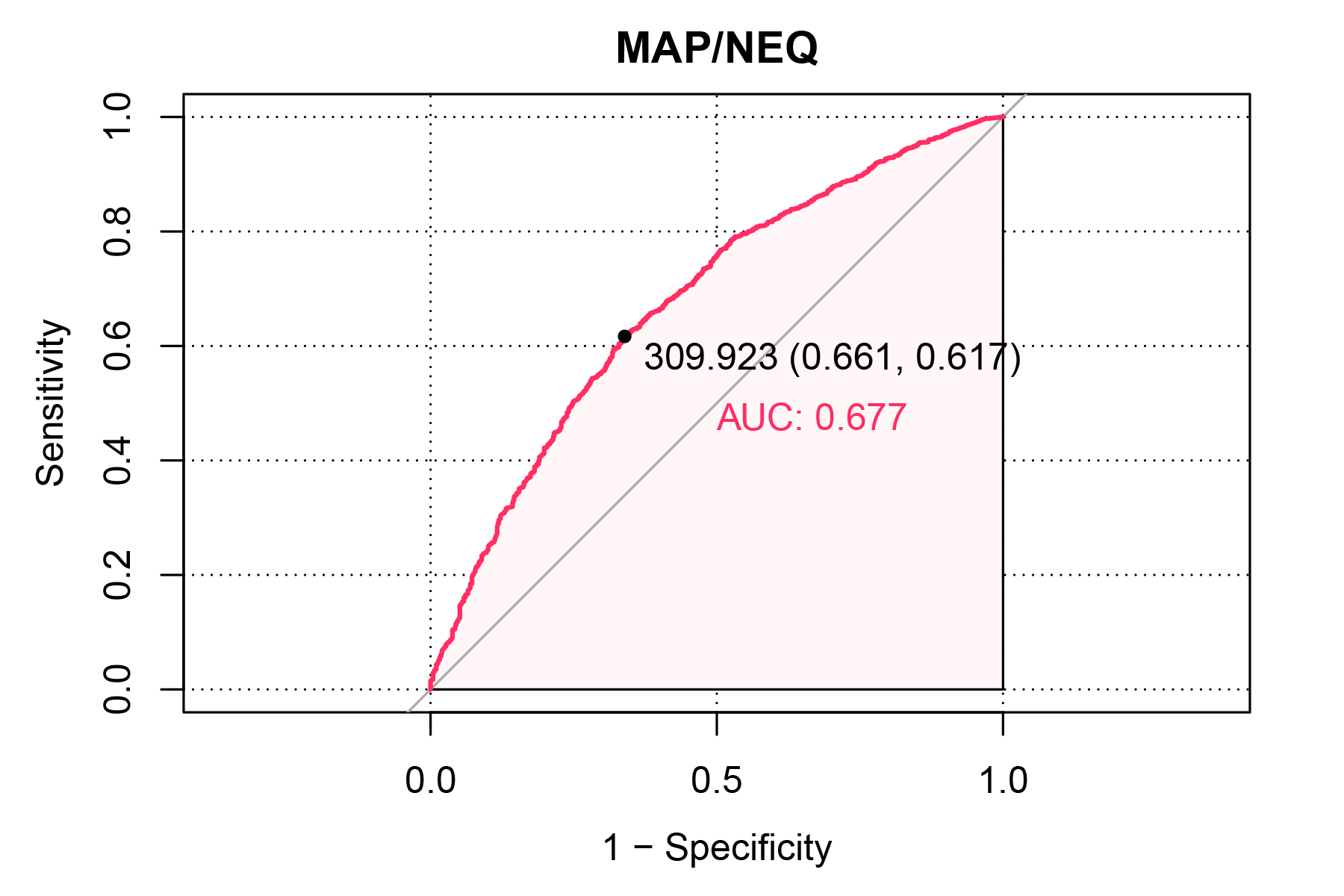

Supplement: Supplementary file 11 — Supplementary file11 (TIF 483 KB) [file 11255_2023_3908_MOESM11_ESM.tif]
