# Supplementary material for: Mean arterial pressure to norepinephrine equivalent dose ratio for predicting renal replacement therapy requirement: a retrospective analysis from the MIMIC-IV
Source: Int Urol Nephrol. 2024 Jan 18;56(6):2065–74. doi: 10.1007/s11255-023-03908-3 (PMC11090965; doi:10.1007/s11255-023-03908-3)
Supplement: Supplementary file 12 — Supplementary file12 (DOC 3125 KB) [file 11255_2023_3908_MOESM12_ESM.doc]

**Supplementary Material**

**Table S1** Outcomes.............................................................................................................................…...2

**Table S2** Baseline characteristics of patients and care processes categorized by sustained renal dysfunction…………………………………………………………………………………………….....3

**Table S3** Maximum rate and average rate of vasopressors within the first day in the ICU………………6

**Table S4** Total amount of vasopressors within the first day in the ICU………………………………....7

**Table S5** The time and stage of AKI based on creatinine, urine volume, and creatinine or urine volume…....................................................................................................................................................8

**Table S6** Univariate logistic regression for RRT and sustained renal dysfunction………………………9

**Table S7** Forward selection logistic regression showing the association between the MAP/NEQ index and sustained renal dysfunction within 30 days………………………………………………………...11

**Fig. S1** Variable selection using least absolute shrinkage and selection operator regression………….12

**Fig. S2** Forest plot showing the relationship between the MAP/NEQ index and RRT in the multivariate logistic regression models...………………………………………….…………………………………13

**Fig. S3** The ROC curve evaluating the predictive ability of the MAP, NEQ, and MAP/NEQ index within the first day of ICU admission to predict RRT within 30 days………………………………....14

**Fig. S4** Forest plot of risk factors for sustained renal dysfunction within 30 days using multivariate logistic regression analysis……………………………………………………………………….……..15

**Fig. S5** The ROC curve evaluating the predictive ability of the MAP, NEQ, and MAP/NEQ index within the first day of ICU admission to predict sustained renal dysfunction within 30 days……........16

**Table S1** Outcomes

| Outcome | All patients  (n=5775) |
| --- | --- |
| RRT | 301/5775 (5.2%) |
| Survivors | 131/4948(2.6%) |
| Non-survivors | 170/827(20.6%) |
| Final serum creatinine level ≥ 200% of the baseline with RRT | 177/5775(3.1%) |
| Survivors | 78/4948 (1.6%) |
| Non-survivors | 99/827(12.0%) |
| Final serum creatinine level ≥ 200% of the baseline without RRT | 528/5775(9.1%) |
| Survivors | 237/4948 (4.8%) |
| Non-survivors | 291/827(35.2%) |

Values were n (%)

*RRT* renal replacement therapy

**Table 2** Baseline characteristics of patients and care processes categorized by sustained renal dysfunction

| Characteristic | Before weighting | | | |  | After weighting | | | |
| --- | --- | --- | --- | --- | --- | --- | --- | --- | --- |
|  | No sustained renal dysfunction (n=4946) | Sustained renal dysfunction (n=705) | *P* | SMD |  | No sustained renal dysfunction (n=630) | Sustained renal dysfunction (n=630) | *P* | SMD |
| Age, years | 64(56-71) | 54(43-71) | 0.029 | -0.082 |  | 64(55-71) | 64(54-71) | 0.927 | 0.011 |
| Male | 3187(64.4%) | 418(59.3%) | 0.008 | -0.105 |  | 367(58.3%) | 366(58.1%) | 0.954 | -0.003 |
| Weight, kg | 84(71-99) | 85(71-102) | 0.096 | 0.083 |  | 85(70-101) | 85(70-102) | 0.909 | 0.000 |
| Baseline creatinine, µmol/L | 61.9(44.2-70.7) | 70.7(44.2-91.2) | <0.001 | 0.214 |  | 61.9(44.2-79.6) | 70.1(44.2-90.0) | 0.247 | 0.011 |
| Hypertension ^a^ | 2688(54.4%) | 330(46.8%) | <0.001 | -0.151 |  | 307(48.7%) | 296(47.0%) | 0.535 | -0.035 |
| Chronic pulmonary disease | 1258(25.4%) | 207 (29.4%) | 0.026 | 0.086 |  | 173(27.5%) | 184(29.2%) | 0.492 | 0.038 |
| Liver disease | 617(12.5%) | 255(36.2%) | <0.001 | 0.493 |  | 210(33.3%) | 193(30.6%) | 0.305 | -0.056 |
| Sepsis shock | 976(19.7%) | 327(46.4%) | <0.001 | 0.534 |  | 262(41.6%) | 272(43.2%) | 0.569 | 0.032 |
| Cardiac shock | 323(6.5%) | 79(11.2%) | <0.001 | 0.148 |  | 74(11.8%) | 72(11.4%) | 0.860 | -0.010 |
| Hemorrhagic shock | 40(0.8%) | 4(0.6%) | 0.495 | -0.032 |  | 6(1.0%) | 4(0.6%) | 0.525 | -0.042 |
| Obstructive shock ^b^ | 167(3.4%) | 30(4.3%) | 0.234 | 0.044 |  | 28(4.4%) | 28(4.4%) | 0.999 | 0.000 |
| Unspecified shock | 3440(69.6%) | 265(37.5%) | <0.001 | 0.197 |  | 260(41.2%) | 254(40.4%) | 0.951 | -0.043 |
| Duration of MAP< 65 mmHg, h |  |  | <0.001 |  |  |  |  | 0.608 |  |
| < 5 | 3400(68.7%) | 413(58.6%) |  | 0.000 |  | 378(60.0%) | 384(61.0%) |  | 0.000 |
| 5-10 | 1015(20.6%) | 162(23.0%) |  | 0.058 |  | 136(21.6%) | 143(22.7%) |  | 0.026 |
| > 10 | 531(10.7%) | 130(18.4%) |  | 0.199 |  | 116(18.4%) | 103(16.3%) |  | -0.053 |
| Fluid Balance, L | 1.69(0.37-3.34) | 3.51(1.44-6.26) | <0.001 | 0.417 |  | 2.85(1.00-5.39) | 3.19(1.19-5.58) | 0.236 | 0.011 |
| Highest chloride level, mmol/L |  |  | <0.001 |  |  |  |  | 0.991 |  |
| <108 | 1633(33.0%) | 160(22.7%) |  | 0.274 |  | 147(23.3%) | 149(23.7%) |  | -0.003 |
| 108-111 | 2113(42.7%) | 397(56.3%) |  | 0.000 |  | 348(55.2%) | 347(55.1%) |  | 0.000 |
| >111 | 1200(24.3%) | 148(21.0%) |  | -0.080 |  | 135(21.5%) | 134(21.2%) |  | -0.004 |
| Inotropic support ^c^ | 316(6.4%) | 65(9.2%) | 0.005 | 0.098 |  | 56(8.9%) | 55(8.7%) | 0.921 | -0.006 |
| Vancomycin administration | 2841(57.4%) | 486(68.9%) | <0.001 | 0.248 |  | 429(68.1%) | 426(67.6%) | 0.856 | -0.010 |
| Red blood cell transfusion | 1248(25.2%) | 230(32.6%) | <0.001 | 0.158 |  | 205(32.5%) | 196(31.1%) | 0.586 | -0.031 |
| Albumin transfusion | 2132(43.1%) | 238(33.8%) | <0.001 | -0.198 |  | 212(33.7%) | 207(32.9%) | 0.765 | -0.017 |
| Furosemide administration | 1374(27.8%) | 159(22.6%) | 0.003 | -0.125 |  | 146(23.2%) | 145(23.0%) | 0.947 | -0.004 |
| Invasive mechanical ventilation | 3561(72.0%) | 582(82.6%) | <0.001 | 0.278 |  | 502(79.7%) | 510(81.0%) | 0.571 | 0.034 |
| Highest lactate level > 2mmol/L | 3400(68.7%) | 518(73.5%) | 0.011 | 0.107 |  | 449(71.3%) | 450(71.4%) | 0.950 | 0.004 |
| Lowest platelet count, ×10^9^/L |  |  | <0.001 |  |  |  |  | 0.956 |  |
| >150 | 2399(48.5%) | 292(41.4%) |  | 0.000 |  | 282(44.8%) | 279(44.3%) |  | 0.000 |
| 100-150 | 1624(32.8%) | 149(21.1%) |  | -0.295 |  | 143(22.7%) | 141(22.4%) |  | -0.020 |
| < 100 | 923(18.7%) | 264(37.5%) |  | 0.388 |  | 205(32.5%) | 210(33.3%) |  | 0.016 |
| MAP/NEQ, mmHg/mcg/kg/min | 401.6(179.9-680.0) | 171.5(11.7-399.7) | <0.001 |  |  | 252.9(14.2-519.0) | 207.0(12.9-432.9) | 0.010 |  |
| SOFA | 7(5-9) | 10(8-13) | <0.001 |  |  | 8(6-11) | 10(8-12) | <0.001 |  |
| APS III | 42(32-58) | 72(53-89) | <0.001 |  |  | 55(40-71) | 69(52-86) | <0.001 |  |
| Congestive heart failure | 1366(27.6%) | 213(30.2%) | 0.151 |  |  | 201(31.9%) | 195(31.0%) | 0.716 |  |
| Diabetes mellitus | 1355(27.4%) | 211(29.9%) | 0.160 |  |  | 169(26.8%) | 195(31.0%) | 0.106 |  |
| Admission type |  |  | <0.001 |  |  |  |  | 0.060 |  |
| Elective | 1787(36.1%) | 128 (18.2%) |  |  |  | 156(24.8%) | 122(19.4%) |  |  |
| Emergency | 2004(40.5%) | 393(55.7%) |  |  |  | 318(50.4%) | 349(55.4%) |  |  |
| Urgent | 1155(23.4%) | 184(26.1%) |  |  |  | 156(24.8%) | 159(25.2%) |  |  |
| MAP ^d^, mmHg | 67(60-74) | 65(58-73) | <0.001 | - |  | 67(60-74) | 66(59-74) | 0.122 |  |
| MAP ^e^, mmHg | 56(50-60) | 53(45-59) | <0.001 | - |  | 54(47-59) | 53(46-59) | 0.314 |  |
| Highest noradrenaline equal rate, μg.kg.min^-1^ |  |  | <0.001 |  |  |  |  | 0.014 |  |
| < 0.10 | 1009(20.4%) | 60(8.5%) |  |  |  | 84(13.3%) | 57(9.0%) |  |  |
| 0.10-0.20 | 1761(35.6%) | 154(21.8%) |  |  |  | 163(25.9%) | 146(23.2%) |  |  |
| > 0.20 | 2176(44.0%) | 491(69.7%) |  |  |  | 383(60.8%) | 427(67.8%) |  |  |
| Cumulative chloride dose, mmol |  |  | <0.001 | - |  |  |  | 0.087 |  |
| < 500 | 2897(58.6%) | 370(52.5%) |  |  |  | 312(49.5%) | 340(54.0%) |  |  |
| 500-1000 | 1692(34.2%) | 243(34.5%) |  |  |  | 220(34.9%) | 217(34.4%) |  |  |
| > 1000 | 357(7.2%) | 92(13.0%) |  |  |  | 98(15.6%) | 73(11.6%) |  |  |

Values were median (IQR) or n (%)

*SMD* standardized mean difference, *MAP* mean arterial pressure, *NEQ* norepinephrine equivalent dose, *SOFA* Sequential Organ Failure Assessment, *APS III* Acute Physiology Score III

^a^ Diagnosis based on the recorded ICD-9 and ICD-10 codes[1]

^b^ Obstructive shock mainly included cardiac tamponade, pulmonary embolism, tension pneumothorax, and aortic dissection

^c^ Inotropic support was defined as the administration of dobutamine or milrinone

^d^ MAP corresponding to the peak rate of vasopressor

^e^ Lowest MAP within 24 hours after ICU admission

**Table S3** Maximum rate and average rate of vasopressors within the first day in the ICU

| Vasopressor | Maximum rate | | | | |  | Average rate | | | | |
| --- | --- | --- | --- | --- | --- | --- | --- | --- | --- | --- | --- |
|  | No RRT  (n=5474)  No. of patients Rate | | RRT  (n=301)  No. of patients Rate | | *P* |  | No RRT  (n=5474)  No. of patients Rate | | RRT  (n=301)  No. of patients Rate | | *P* |
| Norepinephrine | 2961 | 0.20(0.10-0.32) | 267 | 0.35(0.19-0.50) | <0.001 |  | 2961 | 0.10(0.06-0.17) | 267 | 0.20 (0.10-0.31) | <0.001 |
| Epinephrine | 1010 | 0.03(0.03-0.05) | 73 | 0.07(0.04-0.13) | <0.001 |  | 1010 | 0.02(0.02-0.04) | 73 | 0.05(0.03-0.07) | <0.001 |
| Dopamine | 258 | 10.00(5.00-15.03) | 26 | 6.26(5.00-20.00) | 0.407 |  | 258 | 5.93(3.85-9.67) | 26 | 5.18(2.99-12.69) | 0.876 |
| Phenylephrine | 3132 | 1.50(1.00-2.01) | 133 | 2.52(1.81-4.97) | <0.001 |  | 3132 | 0.72(0.48-1.19) | 133 | 1.46(0.89-2.34) | <0.001 |
| Vasopressin | 867 | 2.40(2.40-2.40) | 155 | 2.40(2.40-3.60) | 0.001 |  | 867 | 2.39(2.02-2.41) | 155 | 2.40(2.35-2.43) | <0.001 |

Values were median (IQR)

All values were in mcg/kg/min except vasopressin, which was in units/hour

*ICU* intensive care unit, *RRT* renal replacement therapy

**Table S4** Total amount of vasopressors within the first day in the ICU

| Vasopressor | No RRT  (n=5474) | | | RRT  (n=301) | | | *P* |
| --- | --- | --- | --- | --- | --- | --- | --- |
|  | No. of patients^a^ | Amount ^b^ | % of total amount ^c^ | No. of patients ^a^ | Amount ^b^ | % of total amount ^c^ |  |
| Total amount | 5474 | 80.24(39.29-168.11) | 100% | 301 | 244.60(103.68-481.73) | 100% | <0.001 |
| Norepinephrine | 2961 | 93.18(42.45-188.45) | 53.8% | 267 | 194.92(88.46-394.87) | 65.1% | <0.001 |
| Epinephrine | 1010 | 16.18(8.23-27.92) | 5.0% | 73 | 28.19(8.06-55.40) | 3.9% | 0.004 |
| Dopamine | 258 | 33.97(10.74-73.39) | 1.6% | 26 | 8.94(4.04-76.06) | 1.0% | 0.060 |
| Phenylephrine | 3132 | 51.06(25.05-96.14) | 30.7% | 133 | 76.32(34.91-166.90) | 16.7% | <0.001 |
| Vasopressin | 867 | 75.92(38.30-114.21) | 8.9% | 155 | 88.72(37.30-128.35) | 13.3% | 0.059 |

All values were in mcg/kg except vasopressin, which was in units

*ICU* intensive care unit, *RRT* renal replacement therapy

^a^ Number of patients who received the vasopressor type on each row

^b^ Values were median (IQR)

^c^ Proportion of total vasopressor infusions in the No RRT group and RRT group

**Table S5** The time and stage of AKI based on creatinine, urine volume, and creatinine or urine volume

| Characteristic | Creatinine | | |  | Urine volume | | |  | Creatinine or urine volume | | |
| --- | --- | --- | --- | --- | --- | --- | --- | --- | --- | --- | --- |
|  | No RRT  (n=5474) | RRT  (n=301) | *P* |  | No RRT  (n=5474) | RRT  (n=301) | *P* |  | No RRT  (n=5474) | RRT  (n=301) | *P* |
| Time to first stage of AKI ^a^, day |  |  | <0.001 |  |  |  | 0.329 |  |  |  | 0.084 |
| < 3 | 2137(39.0%) | 274(91.0%) |  |  | 3959(72.3%) | 285(94.7%) |  |  | 4339(79.3%) | 294(97.7) |  |
| 3-7 | 230(4.2%) | 16(5.3%) |  |  | 134(2.4%) | 7(2.3%) |  |  | 129(2.4%) | 4(1.3%) |  |
| > 7 | 122(2.2%) | 2(0.7%) |  |  | 46(0.8%) | 1(0.3%) |  |  | 37(0.7%) | 0(0%) |  |
| First stage of AKI |  |  | 0.701 |  |  |  | <0.001 |  |  |  | 0.029 |
| 1 | 2207(40.3%) | 261(86.7%) |  |  | 3257(59.5%) | 177(58.8) |  |  | 3719(67.9%) | 229(76.1%) |  |
| 2 | 226(4.1%) | 23(7.6%) |  |  | 857(15.7%) | 107(35.5%) |  |  | 728(13.3) | 62(20.6%) |  |
| 3 | 56(1.0%) | 8(2.7%) |  |  | 25(0.5) | 9(3.0%) |  |  | 58(1.1%) | 7(2.3%) |  |
| Time to maximal stage of AKI, day |  |  | <0.001 |  |  |  | <0.001 |  |  |  | 0.021 |
| < 3 | 4987(91.1%) | 209(69.4%) |  |  | 5005(91.4%) | 251(83.4%) |  |  | 4972(90.8%) | 259(76.1%) |  |
| 3-7 | 304(5.6%) | 49(16.3%) |  |  | 312(5.7%) | 29(9.6%) |  |  | 343(6.3%) | 28(20.6%) |  |
| > 7 | 183(3.3%)) | 43(14.3%) |  |  | 157(2.9%) | 21(7.0%) |  |  | 159(2.9%) | 14(2.3%) |  |
| Maximal stage of AKI |  |  | <0.001 |  |  |  | <0.001 |  |  |  | <0.001 |
| 1 | 1542(28.2%) | 40(13.3%) |  |  | 853(15.6%) | 2(0.7%) |  |  | 1063(19.4%) | 1(0.3%) |  |
| 2 | 617(11.3%) | 45(15.0%) |  |  | 2358(43.1%) | 13(4.3%) |  |  | 2389(43.6%) | 11(3.7%) |  |
| 3 | 330(6.0%) | 207(68.8%) |  |  | 928(17.0%) | 278(92.4%) |  |  | 1053(19.2%) | 286(95.0%) |  |

Values were n (%)

*AKI* acute kidney injury, *RRT* renal replacement therapy

^a^ AKI was defined according to the Kidney Disease: Improving Global Outcomes (KDIGO) criteria based on alterations in plasma creatinine and/or oliguria

**Table S6** Univariate logistic regression for RRT and sustained renal dysfunction

| Characteristics | RRT | |  | Sustained renal dysfunction | | Missing number (%) |  |
| --- | --- | --- | --- | --- | --- | --- | --- |
|  | OR(95%CI) | *P* |  | OR(95%CI) | *P* |  |  |
| Age, per 10years | 0.88(0.80-0.95) | 0.002 |  | 0.94(0.88-0.99) | 0.034 | 0(0) |  |
| Male | 0.92(0.72-1.17) | 0.487 |  | 0.80(0.68-0.94) | 0.008 | 0(0) |  |
| Weight, per 10kg | 1.14(1.09-1.19) | <0.001 |  | 1.04(1.01-1.08) | 0.024 | 42(0.7) |  |
| Baseline creatinine, µmol/L | 1.03(1.02-1.04) | <0.001 |  | 1.01(1.00-1.02) | <0.001 | 0(0) |  |
| Hypertension | 0.75(0.60-0.95) | 0.016 |  | 0.74(0.63-0.87) | <0.001 | 0(0) |  |
| Chronic pulmonary disease | 1.20(0.93-1.55) | 0.159 |  | 1.22(1.02-1.45) | 0.026 | 0(0) |  |
| Liver disease | 4.53(3.57-5.77) | <0.001 |  | 3.98(3.34-4.74) | <0.001 | 0(0) |  |
| Congestive heart failure | 1.27(0.99-1.62) | 0.062 |  | 1.13(0.95-1.35) | 0.151 | 0(0) |  |
| Diabetes | 1.08(0.84-1.40) | 0.533 |  | 1.13(0.95-1.35) | 0.160 | 0(0) |  |
| MAP/NEQ, per 100 mmHg/mcg/kg/min | 0.74(0.70-0.78) | <0.001 |  | 0.83(0.81-0.86) | <0.001 | 454(8.1) |  |
| Duration of MAP< 65 mmHg, h |  |  |  |  |  | 0(0) |  |
| < 5 | 1 |  |  | 1 |  |  |  |
| 5-10 | 1.44(1.08-1.91) | 0.012 |  | 1.31(1.08-1.60) | 0.006 |  |  |
| > 10 | 2.16(1.59-2.93) | <0.001 |  | 2.02(1.62-2.50) | <0.001 |  |  |
| Fluid Balance, L | 1.27(1.23-1.31) | <0.001 |  | 1.19(1.16-1.22) | <0.001 | 0(0) |  |
| Highest chloride level, mmol/L |  |  |  |  |  | 1(0.02) |  |
| <108 | 1.69(1.28-2.25) | <0.001 |  | 1.92(1.58-2.33) | <0.001 |  |  |
| 108-111 | 1 |  |  | 1 |  |  |  |
| >111 | 1.13(0.80-1.61) | 0.482 |  | 1.26(0.99-1.59) | 0.056 |  |  |
| Inotropic support ^a^ | 2.68(1.93-3.73) | <0.001 |  | 1.49(1.13-1.97) | 0.005 | 0(0) |  |
| Vancomycin administration | 1.96(1.51-2.55) | <0.001 |  | 1.64(1.39-1.95) | <0.001 | 0(0) |  |
| Furosemide administration | 0.91(0.69-1.18) | 0.471 |  | 0.76(0.63-0.91) | 0.004 | 0(0) |  |
| Invasive mechanical ventilation | 2.75(1.93-3.92) | <0.001 |  | 1.84(1.50-2.26) | <0.001 | 0(0) |  |
| Red blood cell transfusion | 1.78(1.40-2.26) | <0.001 |  | 1.43(1.21-1.70) | <0.001 | 0(0) |  |
| Albumin transfusion | 1.01(0.80-1.28) | 0.907 |  | 0.67(0.57-0.79) | <0.001 | 0(0) |  |
| Highest lactate level > 2mmol/L | 2.79(2.01-3.89) | <0.001 |  | 1.26(1.05-1.50) | 0.011 | 0(0) |  |
| Lowest platelet count, ×10^9^/L |  |  |  |  |  | 11(0.2) |  |
| > 150 | 1 |  |  | 1 |  |  |  |
| 100-150 | 1.10(0.81-1.50) | 0.542 |  | 0.74(0.60-0.91) | 0.004 |  |  |
| < 100 | 3.16(2.42-4.14) | <0001 |  | 2.33(1.95-2.80) | <0.001 |  |  |

*RRT* renal replacement therapy, *OR* odds ratio, *CI* confidence interval, *MAP* mean arterial pressure, *NEQ* norepinephrine equivalent dose

^a^ Inotropic support was defined as the administration of dobutamine or milrinone

**Table S7** Forward selection logistic regression showing the association between the MAP/NEQ index and sustained renal dysfunction within 30 days

| Models | Before weighting  MAP/NEQ index≤365.7 mmHg/mcg/kg/min  n=2763 | | | |  | After weighting  n=1260 | | | |
| --- | --- | --- | --- | --- | --- | --- | --- | --- | --- |
|  | DF | OR (95%CI) | AIC | *P* |  | DF | OR (95%CI) | AIC | *P* |
| Model1 | 2 | 0.72(0.65-0.79) | 2583 | <0.001 |  | 2 | 0.95(0.92-0.98) | 1739 | 0.001 |
| Model2 | 6 | 0.73(0.66-0.80) | 2522 | <0.001 |  | 6 | 0.94(0.92-0.97) | 1747 | 0.001 |
| Model3 | 9 | 0.75(0.68-0.82) | 2415 | <0.001 |  | 9 | 0.94(0.91-0.97) | 1750 | 0.001 |
| Model4 | 14 | 0.77(0.69-0.84) | 2398 | <0.001 |  | 14 | 0.94(0.91-0.97) | 1759 | 0.001 |
| Model5 | 20 | 0.84(0.76-0.93) | 2314 | <0.001 |  | 20 | 0.94(0.91-0.97) | 1770 | 0.001 |

*MAP* mean arterial pressure, NEQ norepinephrine equivalent dose, *DF* degree of freedom, *OR* odds ratio, *CI* confidence interval, *AIC* Akaike’s information criterion

Model 1 was unadjusted

Model 2 was adjusted for age, sex, baseline creatinine

Model 3 was adjusted for Model 2 variables, hypertension, chronic pulmonary disease, and liver disease

Model 4 was adjusted for Model 3 variables, lactate >2 mmol/L, platelet categories, and chloride level

Model 5 was adjusted for Model 4 variables, duration of MAP< 65 mmHg, fluid balance, vancomycin, invasive mechanical ventilation, albumin transfusion


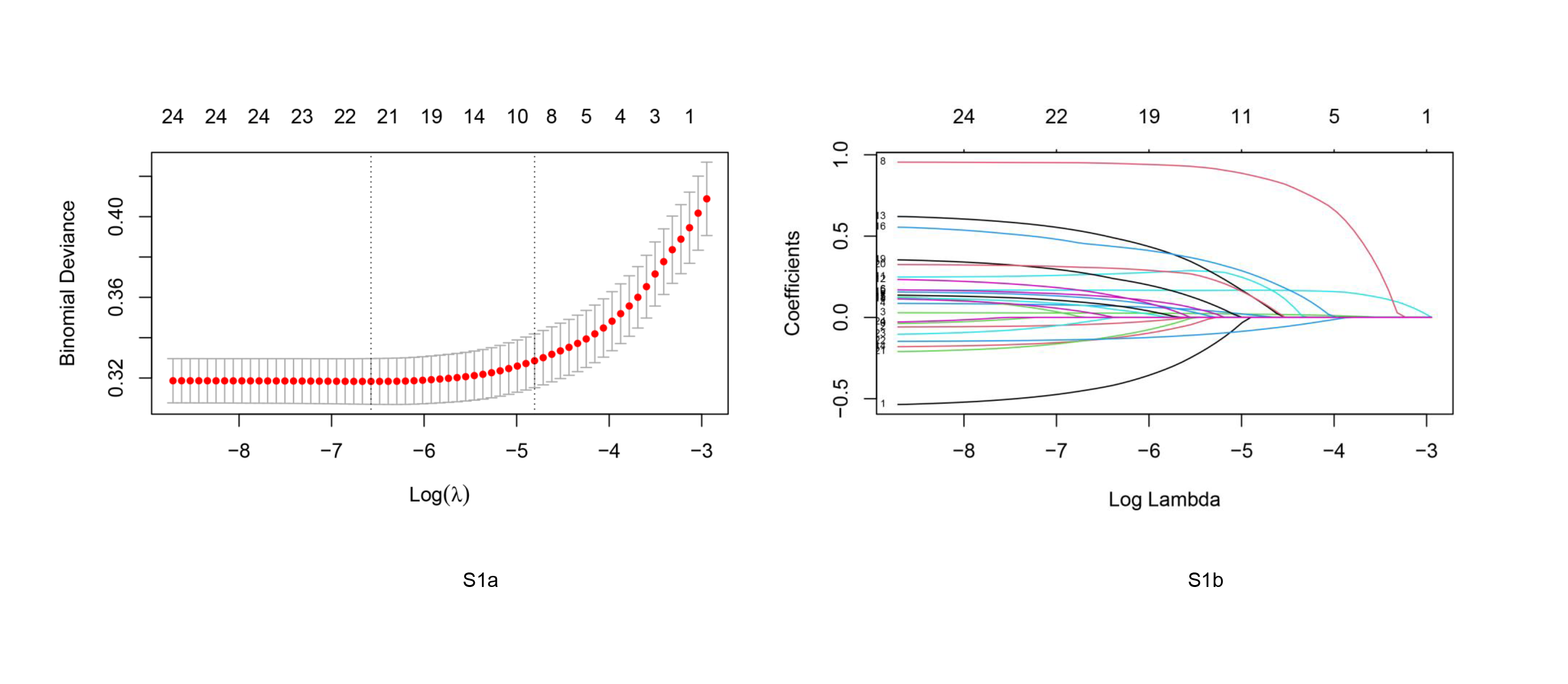


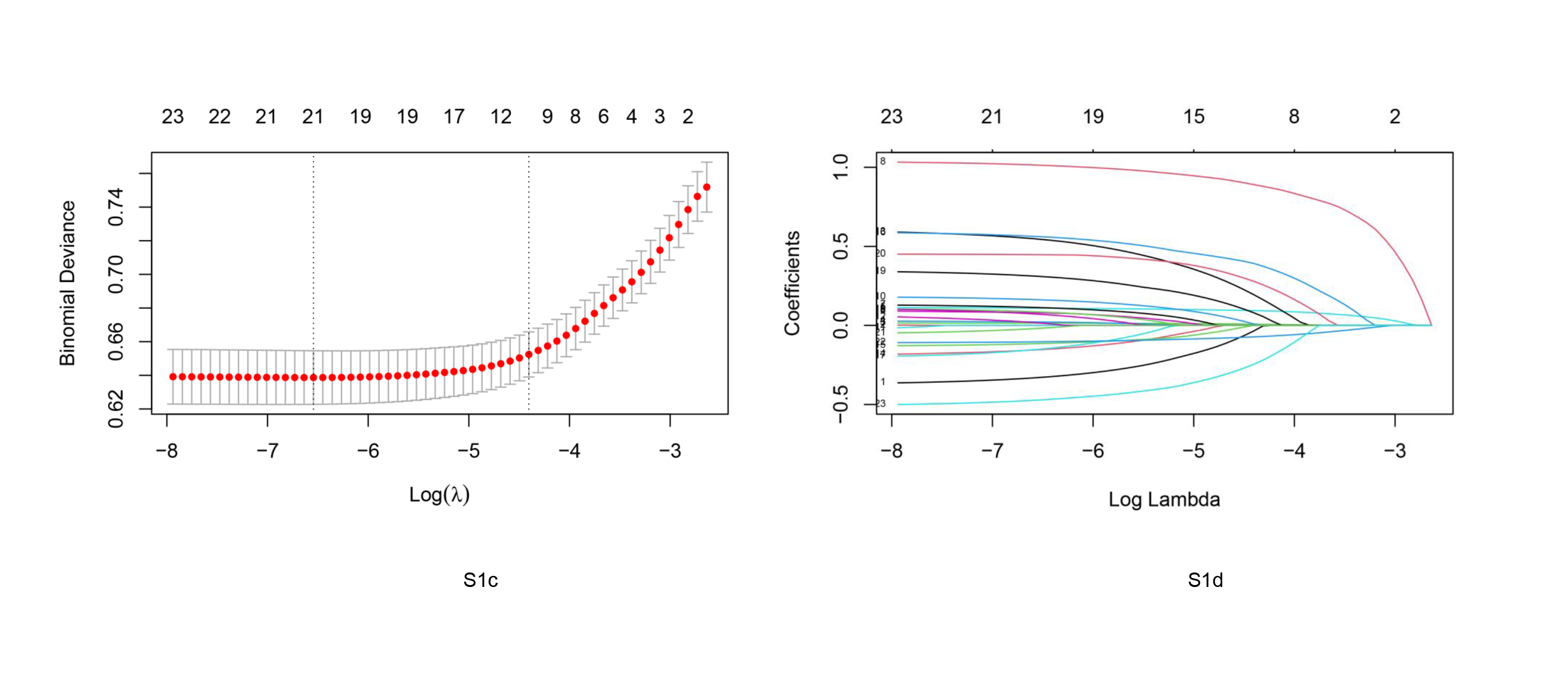


**Fig. S1** Variable selection using least absolute shrinkage and selection operator regression

a, c Plot of binomial deviation versus log (λ) for RRT and sustained renal dysfunction. The optimal tuning parameters (λ) were 0.002 and 0.001 for the variable selection for RRT and sustained renal dysfunction, respectively, when the binomial deviations were minimized. Vertical dashed lines were established at the optimal value of log (λ) for the selected features and one standard error of the minimum value. b, d LASSO coefficient profiles of the variables, each coefficient profile was plotted based on the log (λ) series for RRT and sustained renal dysfunction. A total of 9 variables for RRT and 11 variables for sustained renal dysfunction with non-zero coefficients were reserved.

Variables included the MBP/NEQ index, weight, baseline creatinine, fluid balance, liver disease, invasive mechanical ventilation, highest chloride level, inotropic support, and platelet, which were screened by LASSO regression for RRT. The MBP/NEQ index, gender, baseline creatinine, fluid balance, liver disease, vancomycin administration, albumin transfusion, invasive mechanical ventilation, duration of MAP< 65 mmHg, highest chloride level, and platelet were screened by LASSO regression for sustained renal dysfunction.

*RRT* renal replacement therapy, *LASSO* least absolute shrinkage and selection operator

**
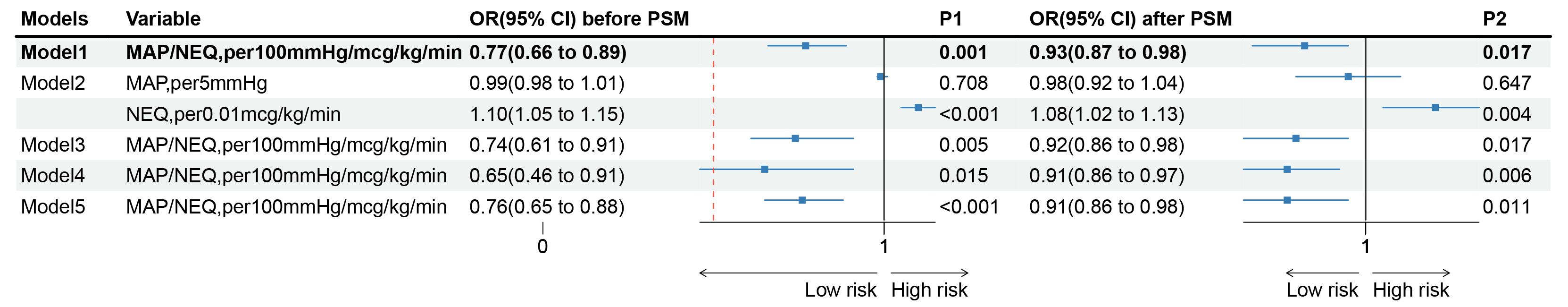
**

**Fig. S2** Forest plot showing the relationship between the MAP/NEQ index and RRT in the multivariate logistic regression models

*OR* odds ratio, *CI* confidence interval, *PSM* propensity score matching, *P1* *P* value before propensity score matching, *P2* *P* value after propensity score matching, *MAP* mean arterial pressure, *NEQ* norepinephrine equivalent dose, *RRT* renal replacement therapy

Model 1 was adjusted the baseline creatinine level for early AKI

Model 2 was adjusted MAP/NEQ index for MAP and NEQ

Model 3 was adjusted MAP corresponding to the peak rate of vasopressor for the lowest MAP within 24 hours

Model 4 was adjusted for patients receiving norepinephrine

Model 5 was adjusted for patients without recorded MAP corresponding to the peak rate of vasopressor


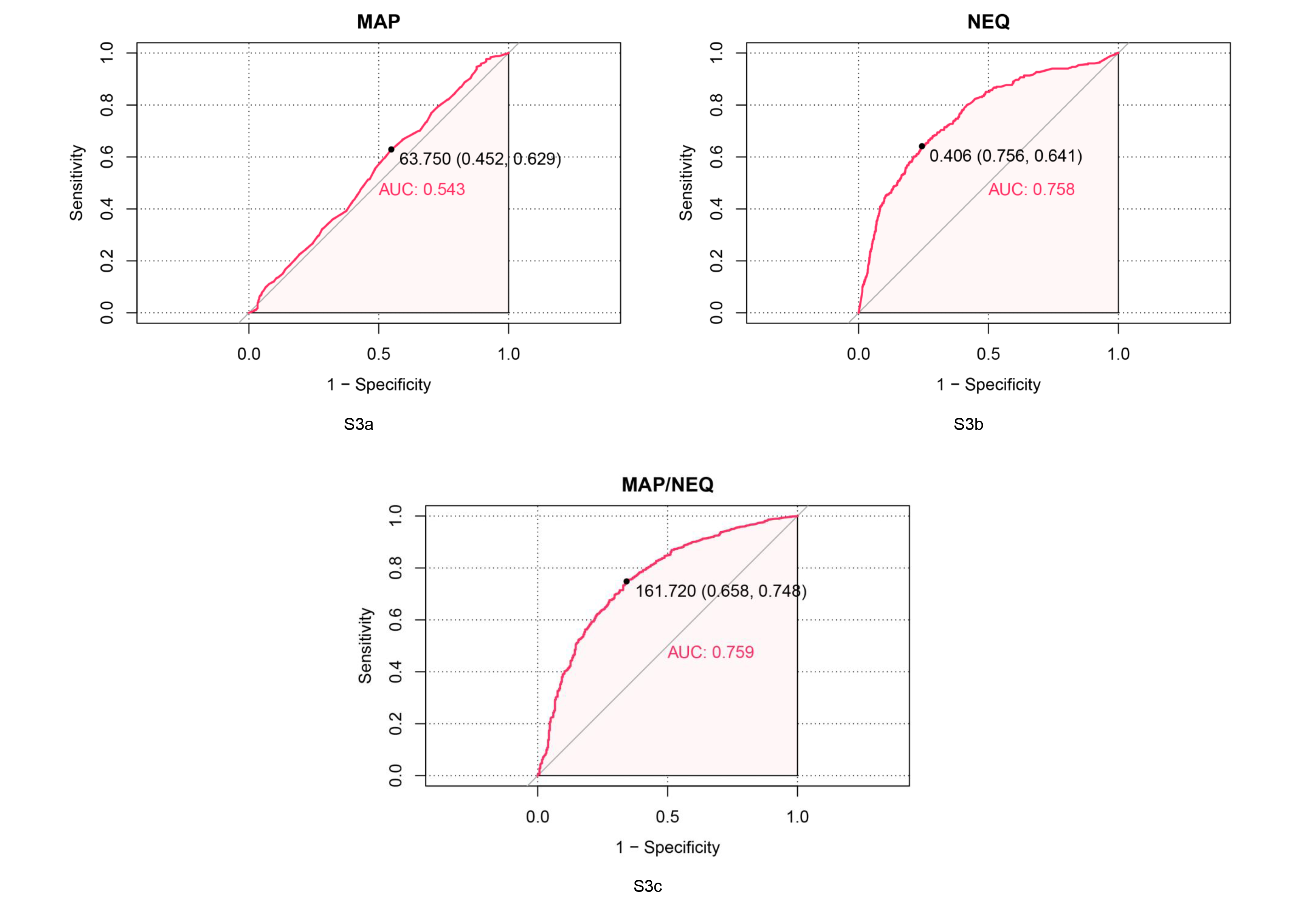


**Fig. S3** The ROC curve evaluating the predictive ability of the MAP, NEQ, and MAP/NEQ index within the first day of ICU admission to predict RRT within 30 days

*MAP* mean arterial pressure, *NEQ* norepinephrine equivalent dose, *ROC* receiver operating characteristic, *ICU* intensive care unit, *RRT* renal replacement therapy

**
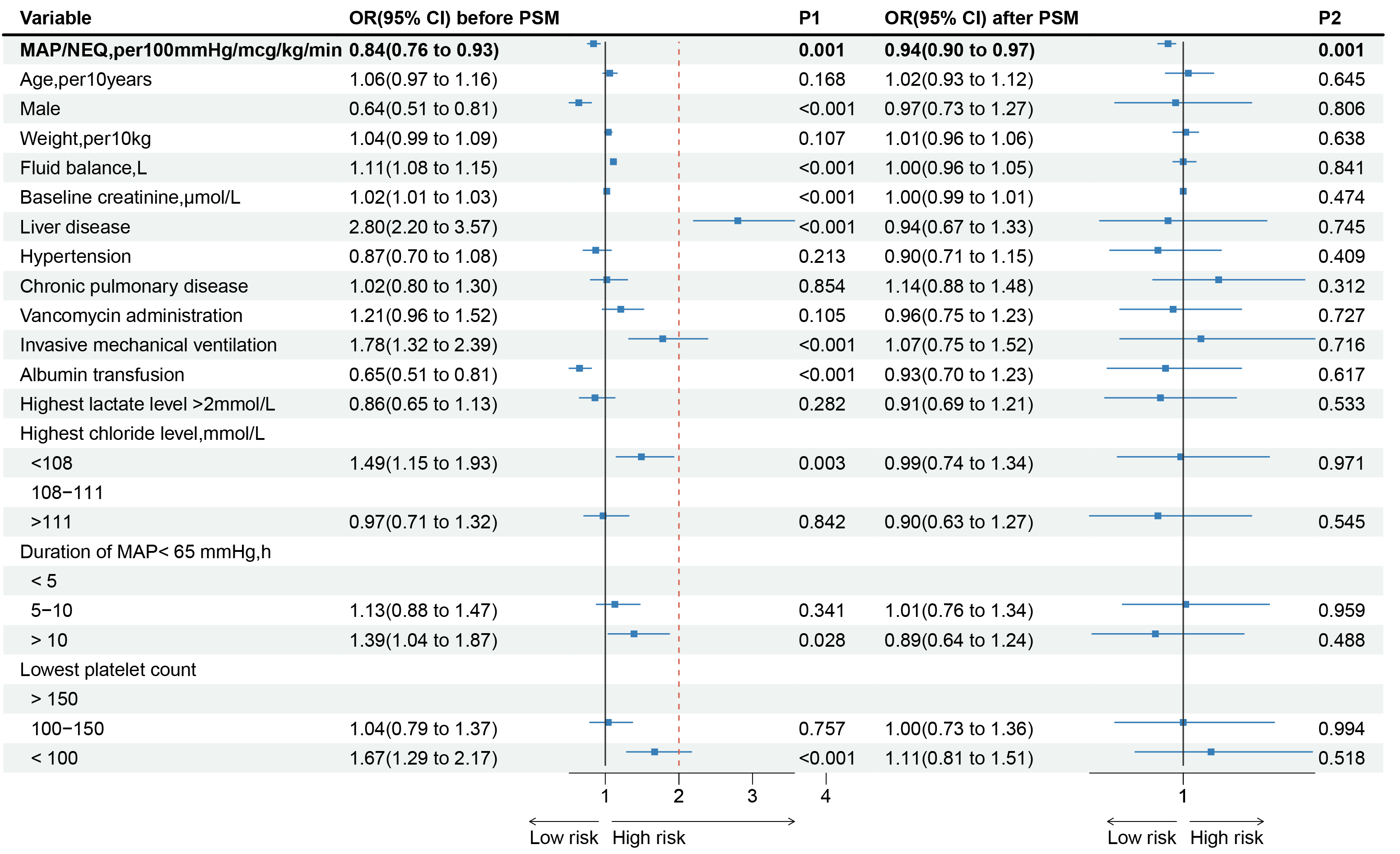
**

**Fig. S4** Forest plot of risk factors for sustained renal dysfunction within 30 days using multivariate logistic regression analysis

*OR* odds ratio, *CI* confidence interval, *PSM* propensity score matching, *P1* *P* value before propensity score matching, *P2* *P* value after propensity score matching, *MAP* mean arterial pressure, *NEQ* norepinephrine equivalent dose


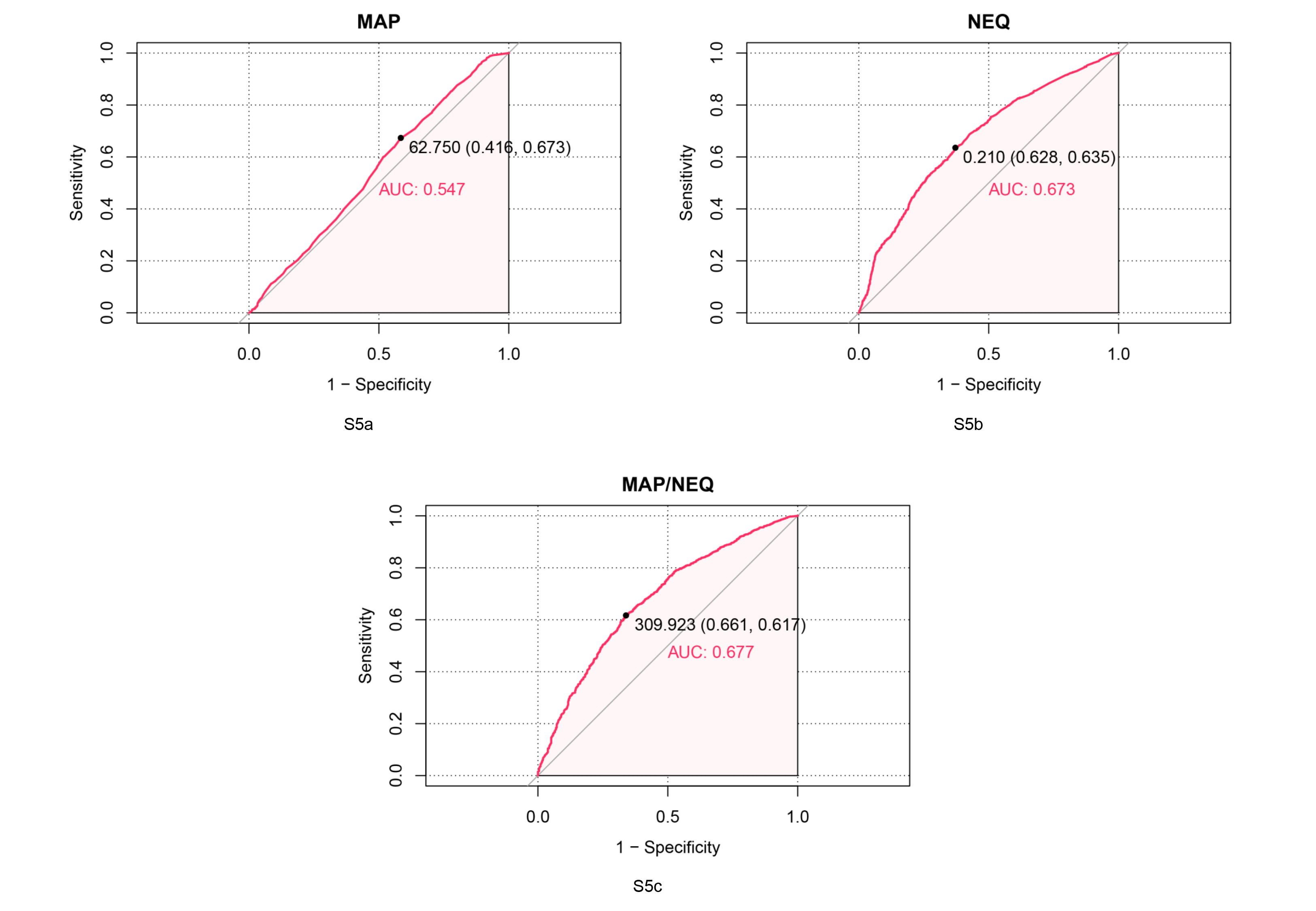


**Fig. S5** The ROC curve evaluating the predictive ability of the MAP, NEQ, and MAP/NEQ index within the first day of ICU admission to predict sustained renal dysfunction within 30 days

*MAP* mean arterial pressure, *NEQ* norepinephrine equivalent dose, *ROC* receiver operating characteristic, *ICU* intensive care unit

**REFERENCE**

1. Johnson AEW, Stone DJ, Celi LA, Pollard TJ (2018) The MIMIC Code Repository: enabling reproducibility in critical care research. J Am Med Inform Assn 25 (1):32-39. doi:10.1093/jamia/ocx084
